# Before the freeze: otoliths from the Eocene of Seymour Island, Antarctica, reveal dominance of gadiform fishes (Teleostei)

**DOI:** 10.1080/14772019.2016.1151958

**Published:** 2016-03-16

**Authors:** Werner Schwarzhans, Thomas Mörs, Andrea Engelbrecht, Marcelo Reguero, Jürgen Kriwet

**Affiliations:** ^a^ Ahrensburger Weg 103, D-22359Hamburg, Germany; ^b^ Natural History Museum of Denmark, Zoological Museum, Universitetsparken 15, DK-2100Copenhagen, Denmark; ^c^ Swedish Museum of Natural History, Department of Palaeobiology, P.O. Box 5007, SE-10405Stockholm, Sweden; ^d^ University of Vienna, Department of Palaeontology, Althanstrasse 14, 1090Vienna, Austria; ^e^ Museo de La Plata, División Paleontología de Vertebrados, Paseo del Bosque s/n, B1900FWALa Plata, Argentina

**Keywords:** temperate climate, *Macruronus*, Macrouridae, Gadiformes, Antarctica, Eocene

## Abstract

The first record of fossil teleostean otoliths from Antarctica is reported. The fossils were obtained from late Early Eocene shell beds of the La Meseta Formation, Seymour Island that represent the last temperate marine climate phase in Antarctica prior to the onset of cooling and subsequent glaciation during the late Eocene. A total of 17 otolith-based teleost taxa are recognized, with 10 being identifiable to species level containing nine new species and one new genus: *Argentina antarctica* sp. nov., *Diaphus*? *marambionis* sp. nov., *Macruronus eastmani* sp. nov., *Coelorinchus balushkini* sp. nov., *Coelorinchus nordenskjoeldi* sp. nov., *Palimphemus seymourensis* sp. nov., *Hoplobrotula*? *antipoda* sp. nov., *Notoberyx cionei* gen. et sp. nov. and *Cepola anderssoni* sp. nov. *Macruronus eastmani* sp. nov. is also known from the late Eocene of Southern Australia, and *Tripterophycis immutatus* Schwarzhans, widespread in the southern oceans during the Eocene, has been recorded from New Zealand, southern Australia, and now Antarctica. The otolith assemblage shows a typical composition of temperate fishes dominated by gadiforms, very similar at genus and family levels to associations known from middle Eocene strata of New Zealand and the late Eocene of southern Australia, but also to the temperate Northern Hemisphere associations from the Paleocene of Denmark. The Seymour Island fauna bridges a gap in the record of global temperate marine teleost faunas during the early Eocene climate maximum. The dominant gadiforms are interpreted as the main temperate faunal component, as in the Paleocene of Denmark. Here they are represented by the families Moridae, Merlucciidae (Macruroninae), Macrouridae and Gadidae. Nowadays Gadidae are a chiefly Northern Hemisphere temperate family. Moridae, Macruroninae and Macrouridae live today on the lower shelf to deep-water or mesopelagically with Macruroninae being restricted to the Southern Ocean. The extant endemic Antarctic gadiform family Muraenolepididae is missing, as are the dominant modern Antarctic fishes of the perciform suborder Notothenioidei. Recently, there has been much debate on isolated jaw bones of teleost fishes found in the La Meseta Formation and whether they would represent gadiforms (Merlucciidae in this case) or some early, primitive notothenioid. Otoliths are known to often complement rather than duplicate skeletal finds. With this in mind, we conclude that our otolith data support the presence of gadiforms in the early Eocene of Antarctica while it does not rule out the presence of notothenioids at the same time.

http://zoobank.org/urn:lsid:zoobank.org:pub:A30E5364-0003-4467-B902-43A41AD456CC

## Introduction

Few fossil fish remains have been recovered from the Cenozoic of Antarctica, partly because outcrops are scarce due to ice coverage, and also because Cenozoic strata were rarely identified along the islands of the Antarctic Peninsula. The Eocene La Meseta Formation on Seymour Island represents the prime location for Palaeogene fish fossils in Antarctica. Previous studies have documented isolated bones, teeth and scales (Grande & Eastman 1986; Eastman & Grande 1989, 1991; Long 1991, 1992; Jerzmanska & Swidnicki 1992; Balushkin 1994; Cione *et al.* 1994; Doktor *et al*. 1996; Long & Stiwell 2000; Kriwet & Hecht 2008; Claeson *et al*. 2012; Bienkowska-Wasiluk *et al*. 2013;) and in one case articulated skeletons of a clupeid – *Marambionella andreae* Jerzmanska, 1991. Disarticulated fish remains have been identified as representing gadiforms, mostly of the families Merlucciidae (Eastman & Grande 1989; Jerzmanska & Swidnicki 1992; Long & Stiwell 2000; Claeson *et al.* 2012) and Macrouridae (Kriwet & Hecht 2008), beryciforms (Doktor *et al.* 1996), and perciforms of the families Oplegnathidae (Cione *et al.* 1994), Labridae (Long 1992), Trichiuridae (Long 1991) and Notothenioidei (Balushkin 1994; Bienkowska-Wasiluk *et al.* 2013). Much debate has developed around the nature of certain fish remains considered to represent gadiforms, Merlucciidae in this case (Eastman & Grande 1991; Claeson *et al.* 2012) or, alternatively, primitive notothenioids like *Proeleginops grandeastmanorum* Balushkin, 1994 and *Mesetaichthys jerzmanskae* Bienkowska-Wasiluk, Bonde, Møller and Gazdzicki, 2013.

Here, we report fossil otoliths obtained from the Telm 5 interval of Late Ypresian age (see below) representing the first fossil otoliths described from Antarctica. Otoliths were relatively few and had suffered from exposure to the harsh Antarctic conditions. Periodic thawing of the outcrop surface often results in moisture invading the aragonitic inter-crystal structure of the otoliths and subsequent freezing results in the cracking of such specimens. Therefore, the otolith material we obtained consists of many fragmented specimens or specimens with variable degrees of surface leaching and erosional effects. A typical feature of leaching and erosion, for instance, is relief reversal along the otolith rims, where furrows at the otolith margins acted as hard and stiff loci, resistant to erosion while the lobes between the furrows were softened and then eroded. Other effects of leaching and chemical reactions with the surrounding sediment are the formation of incrustations across part of the otolith or damage of selected surface structures. As a result, only 65 of our otolith specimens are identifiable to genus or higher taxonomic level and only 52 specimens to the species level. Fewer still are considered to be suitable to serve as types. However, most specimens retrieved are of a comparatively large size and generally reflect diagnostic maturation and show sufficient characters for definition, when reasonably preserved.

Despite all these caveats and restrictions, the obtained otoliths greatly increase the number of teleostean taxa now recorded from the Eocene of Antarctica. A total of 17 otolith-based taxa are recognized, representing 14 different families within the orders Argentiniformes, Aulopiformes, Myctophiformes, Gadiformes, Ophidiiformes, Beryciformes and Perciformes, none of which however represent any of the extant sub-Antarctic fishes, such as the Notothenioidei or Muraenolepididae. Nine species are described as new to science, one species is identical with records from the Eocene of New Zealand and South Australia, and seven species remain in open nomenclature.

## Geological setting

**Figure 1.**
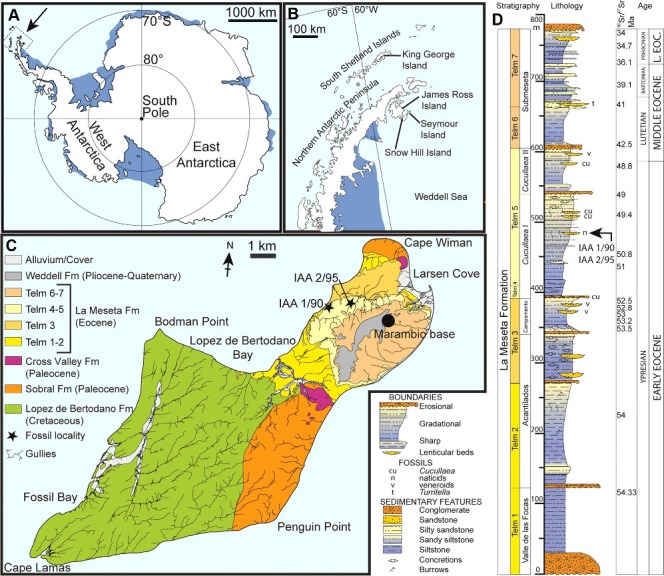
Location and stratigraphy. **A,** map of Antarctica showing the position of the Antarctic Peninsula; **B,** map of the Antarctic Peninsula showing Seymour Island; **C,** geological map of Seymour Island showing the outcrop of Telm 4-5 and localities IAA 1/90 and 2/95; **D,** composite measured section through the La Meseta Formation showing the stratigraphical position of the sampled ‘*Natica* horizon’ (IAA 1/90 and 2/95). Modified from Reguero *et al*. (2013). Strontium date values from Dutton *et al*. (2002), Ivany *et al*. (2008), Dingle & Lavelle (1998) and Reguero *et al*. (2002).

Eocene strata exposed on Seymour Island (Fig. 1) are part of the sediment fill of the James Ross Basin, a back-arc basin situated on the eastern (Weddell Sea) flank of the Antarctic Peninsula (Del Valle *et al.* 1992; Elliot 1988; Hathway 2000). The basin covers an area of more than 10,000 km^2^ and is the only place in Antarctica with Palaeogene exposures. The early to late Eocene/?earliest Oligocene La Meseta Formation (Fig. 1) (Elliot & Trautman 1982; Ivany *et al.* 2006) rests unconformably on either Late Cretaceous or Paleocene units and was deposited in deltaic, estuarine and shallow marine environments, filling up a north-west to south-east trending incised valley (Stilwell & Zinsmeister 1992; Marenssi *et al.* 1998, 2002). The La Meseta Formation is composed of poorly consolidated sandstones and mudstones with interbedded shell-rich conglomerates with a composite thickness of 720 metres.

**Figure 2.**
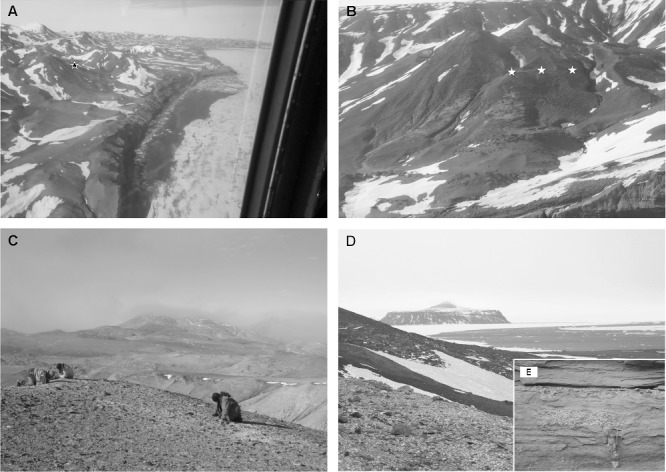
Location photographs. **A,** aerial view of IAA 1/90, ‘Ungulate site’, 64°14′04.67″S,56°39′56.38″ W, marked by asterisk; **B,** panoramic view of site IAA 1/90 with ‘*Natica* horizon’ marked by asterisks; **C,** Argentine-Swedish field party collecting fossils at IAA 2/95, ‘Marsupial site’, 64°13′58″S,56°39′06″ W); **D,** panoramic view of site IAA 2/95 with Cockburn Island in background; **E,** ‘*Natica* horizon’ near site IAA 2/95 showing lens-like character of the beds. Photographs by F. Degrange (A, D), T. Mörs (B) and J. Hagström (C, E).

The majority of otoliths described here come from Seymour Island localities IAA 1/90 (Figs 1, 2A, B; also known as the ‘Ungulate site’; 64° 14′ 04.67″ S, 56° 39′ 56.38″ W) and IAA 2/95 (Figs 1, 2C, D; also known as the ‘Marsupial site’; 64° 13′ 58″ S, 56° 39' 06″ W). Both sites expose lenses of a shelly conglomerate at the same stratigraphical level within the Telm 5 unit that is informally referred to as the ‘*Natica* horizon’ (Fig. 2E), because the bioclast component of the fine to coarse sediment matrix is dominated by the naticid gastropod *Polynices*. Both sites are situated within the *Struthiolarella steinmanni* Zone of Stilwell & Zinsmeister (1992). The ‘*Natica* horizon’ occurs in the central portion of the Cucullaea I Allomember of the La Meseta Formation, within unit Telm 5 of Sadler (1988).

The depositional setting of the *Cucullaea* I Allomember is interpreted as a nearshore, ebb-tidal delta-barrier island complex, strongly influenced by waves and tidal currents (Stilwell & Zinsmeister 1992). Its fossil content is very much restricted to the shell beds and lenses. It is dominated by a few marine mollusc species, like the large, thick-shelled bivalve *Cucullaea raea* and the thick-shelled gastropods *Antarctodarwinella nordenskjoldi* and *Struthiolarella steinmanni*, which are associated with other marine molluscs (for other taxa, see Stilwell & Zinsmeister 1992). Additional marine invertebrates are brachiopods, crinoids, starfish, asteroids, sea urchins, nautiloids and sand crabs. Trace fossils are abundant and include *Ophiomorpha*, *Scoyenia*, *Diplocraterion* and *Skolithos* (Cione & Reguero 1998). Marine vertebrates are also abundant, especially teeth and placoid scales of sharks, skates, rays and chimaeras. With at least 21 taxa representing 11 families, the selachian fauna is more diverse than most extant cool temperate shark faunas and nearly equal to present-day tropical shark faunas (Reguero *et al.* 2013). Additionally, there are teeth and bones of marine teleosts and basilosaurid whales, turtle plates and bones of penguins. Fossil wood is usually heavily bored by teredinid bivalves.

There is a consensus that the age of the Cucullaea I Allomember is Eocene, but there is still a debate whether it is early, middle or even late Eocene. Stilwell & Zinsmeister (1992) suggested a late Eocene age based on struthiolariid gastropods. Strontium isotope ratios (^87^Sr/^86^Sr) from bivalve shells were used by Dutton *et al.* (2002) to argue for a middle Eocene age (44.5 or 47.4 Ma). Strontium data used by Ivany *et al.* (2008) resulted in a late Early Eocene age (49–51 Ma). This older setting is confirmed by Gelfo *et al.* (2009) and Tejedor *et al.* (2009) who correlated the mammal assemblage from Cucullaea I with the Patagonian locality Paso del Sapo, resulting in a latest Early–earliest Middle Eocene age (∼49.5 Ma). In contrast, Douglas *et al.* (2014) proposed an age younger than late Middle Eocene (∼41 Ma) for Telm 5 on the basis of the dinoflagellate cyst assemblage. According to the chronostratigraphical synthesis by Montes *et al.* (2013), the Cucullaea I Allomember has a basal age of 52.8 Ma and a top age of 49.0 Ma, resulting in a late Early Eocene, Ypresian age, an age which is accepted here (Fig. 1).

## Material and methods

The otolith material described here was obtained from sediment samples that were collected during a joint Argentine/Swedish field project during the austral summers 2011 to 2013. Sediment samples were dry-sieved in the field using screens with a 2 cm mesh in order to produce a residue without larger stones and large fragments of bivalves and gastropods. The residue was dry-sieved in the laboratory and separated into finer fractions > 4.0, > 2.0 and > 0.5 mm in order to collect micro-vertebrates. The otoliths were hand-picked using a stereomicroscope.

**Figure 3.**
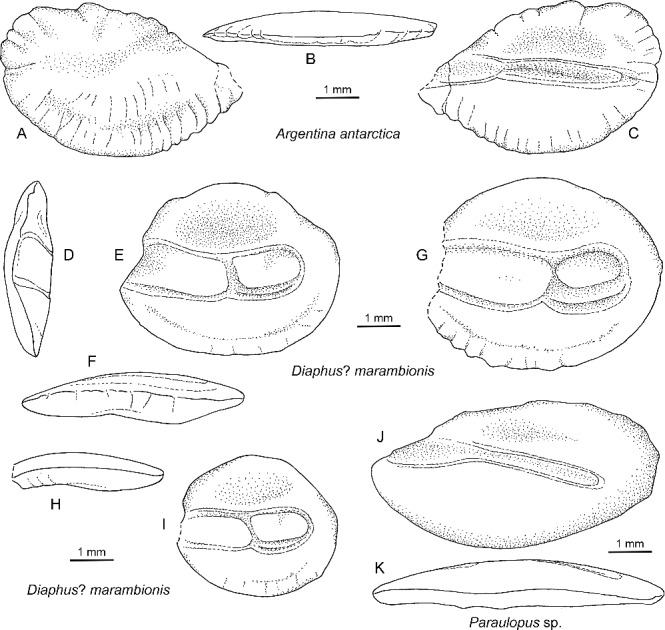
Drawings of Eocene otoliths from Seymour Island. **A–C,** *Argentina antarctica* sp. nov., holotype, NRM-PZ P.15964, mirror imaged; **A,** outer face; **B,** ventral view; **C,** inner face. **D–I,** *Diaphus*? *marambionis* sp. nov.; **D–F,** holotype, NRM-PZ P.15966; **D,** anterior view, **E,** inner face, **F,** ventral view; **G–I,** (mirror imaged) paratypes, NRM-PZ P.15967; **G,** inner face; **H,** ventral view; **I,** inner face. **J, K,** *Paraulopus* sp., NRM-PZ P.15965, mirror imaged; **J,** inner face; **K,** ventral view.

**Figure 4.**
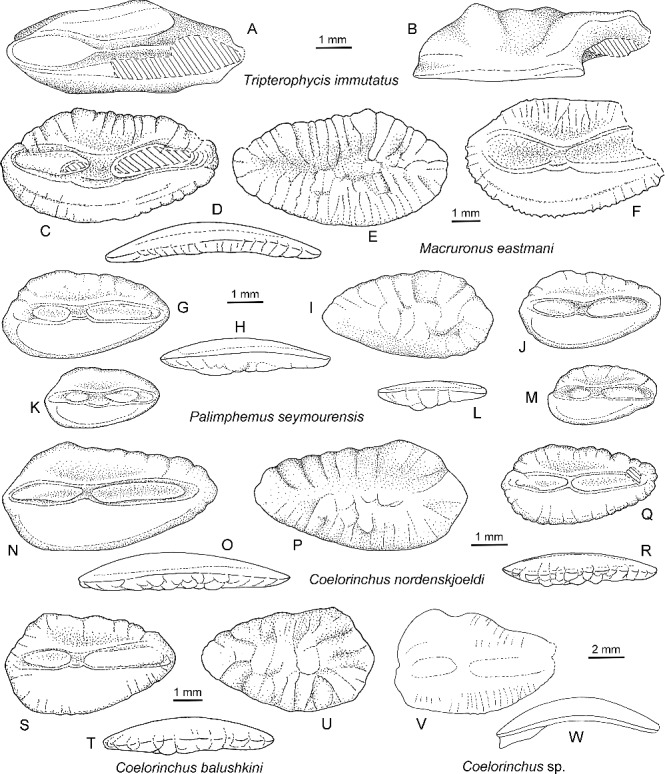
Drawings of Eocene otoliths from Seymour Island. **A, B,** *Tripterophycis immutatus* Schwarzhans, 1980, NRM-PZ P.15969, mirror imaged; **A,** inner face; **B,** dorsal view. **C–F,** *Macruronus eastmani* sp. nov.; **C–E,** holotype, NRM-PZ P.15970, mirror imaged; **C,** inner face; **D,** outer face; **E,** ventral view; **F,** paratype, NRM-PZ P.15971, inner face. **G–M,** *Palimphemus seymourensis* sp. nov.; **G–I,** holotype, NRM-PZ P.15973, mirror imaged; **G,** inner face; **H,** ventral view; **I,** outer face; **J,** paratype, NRM-PZ P.15975, mirror imaged, inner face; **K–M,** paratypes, NRM-PZ P.15974; **K,** inner face, mirror imaged; **L,** ventral view; **M,** inner face. **N–R,** *Coelorinchus nordenskjoeldi* sp. nov.; **N–P,** holotype, NRM-PZ P.15978; **N,** inner face; **O,** ventral view; **P,** outer face; **Q, R,** paratype, NRM-PZ P.15979, mirror imaged; **Q,** inner face; **R,** ventral view. **S–U,** *Coelorinchus balushkini* sp. nov., holotype, NRM-PZ P.15976; **S,** inner face; **T,** ventral view; **U,** outer face. **V, W,** *Coelorinchus* sp., NRM-PZ P.15911; **V,** inner face (strongly eroded); **W,** ventral view.

**Figure 5.**
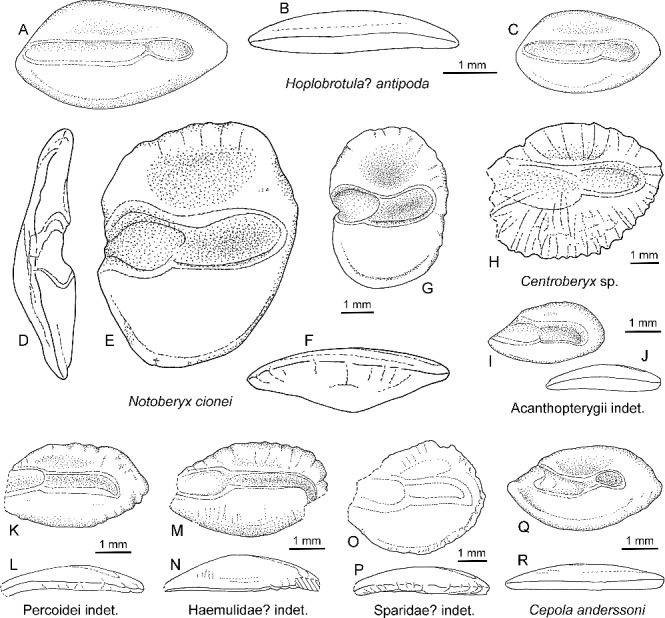
Drawings of Eocene otoliths from Seymour Island. **A–C,** *Hoplobrotula*? *antipoda* sp. nov.; **A, B,** holotype, NRM-PZ P.15984, mirror imaged; **A,** inner face; **B,** ventral view; **C,** paratype, NRM-PZ P.15985, inner face. **D–G,** *Notoberyx cionei* gen. nov., sp. nov.; **D–F,** holotype, NRM-PZ P.15987; **D,** anterior view; **E,** inner face; **F,** ventral view; **G,** paratype, NRM-PZ P.15988, inner face. **H,** *Centroberyx* sp., NRM-PZ P.15986, mirror imaged, inner face. **I, J,** Acanthopterygii indet., NRM-PZ P.15990, mirror imaged; **I,** inner face, **J,** ventral view. **K, L,** Percoidei indet., NRM-PZ P.15992, mirror imaged; **K,** inner face; **L,** ventral view. **M, N,** Haemulidae? indet., NRM-PZ P.15993, mirror imaged; **M,** inner face; **N,** ventral view. **O, P,** Sparidae? indet., NRM-PZ P.15994, mirror imaged; **O,** inner face; **P,** ventral view. **Q, R,** *Cepola anderssoni* sp. nov., holotype, NRM-PZ P.15996, mirror imaged; **Q,** inner face; **R,** ventral view.

Due to the adverse weathering effects on the otolith morphology, satisfactory photographs are difficult to obtain. We have restricted photographs to holotypes mainly for documentary purposes. A more extensive documentation is represented by drawings of the otolith specimens undertaken with a camera lucida (Figs 3–5), in which interpolation can be used to reconstruct areas affected by weathering, minor damage or surface encrustations. Hatched areas in the drawings mark larger areas of damage that cannot be reconstructed.

The otoliths are deposited in the palaeo-zoological collections of the Swedish Museum of Natural History, Stockholm, under the collection registration numbers NRM-PZ P.15911, 15964–15998.

The terminology employed here for the morphological description of the otoliths follows Koken (1891), Weiler (1942) and Schwarzhans (1978). The morphometric measurements follow Schwarzhans (2013). The following abbreviations are used: **OL**, otolith length; **OH**, otolith height; **OT**, otolith thickness; **OsL**, ostium length; **CaL**, cauda length; **OCL**, ostial colliculum length; **CCL**, caudal colliculum length; **OsH**, ostium height; **CaH**, cauda height; **SuL**, sulcus length. The caudal curvature index was calculated as the ratio of the horizontal stretch of the cauda against the length of the inclined portion and was manually measured along the respective ventral and anterior margins of the cauda from the inclination point. The rostrum length was measured from the tip of the rostrum to the level of the deepest point of incision of the excisura, or, in the absence of an excisura, to the point where the dorsal margin of the ostium meets the otolith rim, and was calculated as percentage of OL.

All otoliths are shown as if from the right side in order to facilitate easier comparison. Left otoliths are mirror imaged and annotated accordingly in the figure captions.

## Systematic palaeontology

Fossil skeletons of teleost fishes from the Palaeogene are mostly referred to extinct genera (see for instance Bannikov 2010), while Palaeogene otoliths are often associated with extant genera. We believe that this apparent discrepancy is mainly due to two reasons. Firstly, fish skeletons offer many more characters for diagnoses than otoliths, which makes it easier for palaeoichthyologists dealing with articulated skeletons to recognize diagnostic differences at higher systematic levels; and, secondly, otolith palaeontologists tend to be very conservative when it comes to genus-level taxonomy mainly to avoid establishment of excessive otolith-based fossil genera or alternatively to avoid usage of open generic nomenclature.

Names in so-called open generic nomenclature in otoliths were constructed in the past by applying genitive plural forms of the family or higher taxonomic unit into which the fossil otolith-based species could be placed with comfort by the authors, for example *Otolithus* (Albulidarum) *circularis* following Koken (1884) or ‘genus Albulidarum’ *circularis* following Nolf (1985) for a given species considered to be an albulid of unknown generic relationship. This practice has long been known not to be compliant with the regulations of the ICZN (see Schwarzhans (2012) for a detailed discussion). Recently, Nolf (2013) reviewed this practice and proposed an alternative scheme – ‘*Albulida*’ *circularis*, meaning ‘an albulid’ of unknown generic relationship. In a book review of Nolf's work, Tracey (2014) concluded that this new practice would have to be considered as ‘zoological formulae’ according to article 1.3.7 of the ICZN. We conclude that the methods proposed by Nolf would not be compliant with the ICZN and have therefore refrained from employing these practices in cases of unresolved generic allocations, and instead we follow Janssen (2012) by using an unambiguous genus name followed by a question mark.

The classification below follows Nelson (2006).

Class **Osteichthyes** Huxley, 1880

Division **Teleostei** Müller, 1846

Order **Argentiniformes** Bertelsen, 1958

Suborder **Argentinoidei** Bertelsen, 1958

Family **Argentinidae** Bonaparte, 1846

Genus ***Argentina*** Linnaeus, 1758

***Argentina antarctica*** sp. nov.

(Figs 3A–C, 6A)

#### Holotype

**Figure 6.**
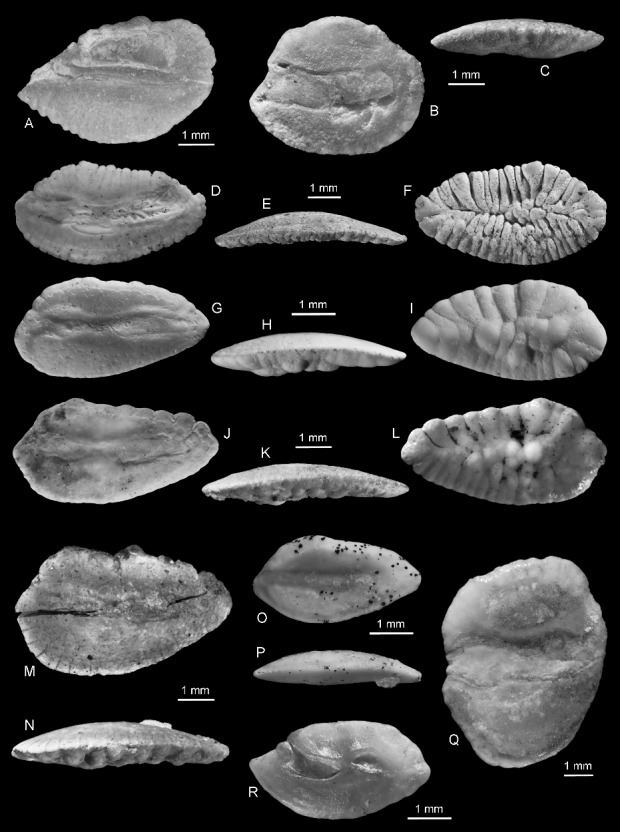
Eocene otoliths from Seymour Island. **A,** *Argentina antarctica* sp. nov., holotype, NRM-PZ P.15964, mirror imaged, inner face. **B, C,** *Diaphus*? *marambionis* sp. nov., holotype, NRM-PZ P.15966; **B,** inner face; **C,** ventral view. **D–F,** *Macruronus eastmani* sp. nov., holotype, NRM-PZ P.15970, mirror imaged; **D,** inner face; **E,** ventral view; **F,** outer face. **G–I,** *Palimphemus seymourensis* sp. nov., holotype, NRM-PZ P.15973, mirror imaged; **G,** inner face; **H,** ventral view; **I,** outer face. **J–L,** *Coelorinchus nordenskjoeldi* sp. nov., holotype, NRM-PZ P.15978; **J,** inner face; **K,** ventral view; **L,** outer face. **M, N,** *Coelorinchus balushkini* sp. nov., holotype, NRM-PZ P.15976; **M,** inner face; **N,** ventral view. **O, P,** *Hoplobrotula*? *antipoda* sp. nov., holotype, NRM-PZ P.15984, mirror imaged; **O,** inner face; **P,** ventral view. **Q,** *Notoberyx cionei* gen. nov., sp. nov.; holotype, NRM-PZ P.15987, inner face. **R,** *Cepola anderssoni* sp. nov., holotype, NRM-PZ P.15996, mirror imaged, inner face.

NRM-PZ P.15964 (Figs 3A–C, 6A) (only specimen).

#### Occurrence

Telm 5 unit; ‘*Natica* horizon’, *Cucullea* I member, La Meseta Formation, late Ypresian, early Eocene. Site IAA 2/95, Seymour Island, Antarctica.

#### Etymology

Named after its occurrence in the Eocene of Antarctica.

#### Diagnosis

Dorsal rim anteriorly depressed, without excisura; posteriorly broadly expanded. Ventral rim deep and regularly curved. All rims intensely crenulated. Cauda straight, terminating close to posterior rim of otolith, not connected through postcaudal depression.

#### Description

A single, thin, large otolith of 5.6 mm in length, mostly well preserved except for the anterior tip of the rostrum. OL:OH = 1.6; OH:OT = 4.3. Dorsal rim anteriorly depressed, nearly straight, inclined, without excisura at upper rim of ostial opening; posteriorly much expanded with broadly rounded postdorsal angle and faint denticle at middorsal position. Ventral rim deeply and regularly curved, deepest at about the middle. Rostrum triangular in shape, moderately long and thin, its ultimate tip broken off. Posterior rim irregularly rounded, dorsally shifted. All rims intensely crenulated, dorsal rim more coarsely than ventral rim, the latter particularly narrowly crenulated anteriorly at rostrum.

Inner face almost flat, with narrow, slightly supramedian, moderately deep sulcus. Ostium anteriorly damaged, originally probably half as long as cauda, somewhat widened; cauda long, narrow, moderately deep, slightly widened at its central section and slightly inclined, terminating close to posterior rim of otolith but not connected via postcaudal depression. Dorsal depression wide; dorsal field with short radial furrows from crenulation of rims; ventral field smooth, without ventral furrow but several short radial furrows particularly along ventral margin of rostrum. Outer face flat with many radial furrows on its ventral half.

#### Remarks

Argentinid otoliths are common in the temperate to cool realms of the Paleocene and early Eocene of the North Atlantic. Four species have been recorded from the Paleocene of Denmark (Schwarzhans 2003), two from the late Paleocene to early Eocene of England (Stinton 1965, 1966) (see revision in Nolf 2013), one from a similar time interval of Ellesmere Island (Schwarzhans 1986) and one from the Paleocene of West Greenland (Schwarzhans 2004). The four species involved are:

*Argentina tricrenulata* (Stinton, 1965). The name *A. tricrenulata* has gained priority over *A. erratica* (Roedel, 1930) *sensu* Schwarzhans (2003) after Nolf (2013) assigned a lectotype representing a different species (see below). Other synonymies listed in Schwarzhans (2003) for *A. erratica* remain with *A. tricrenulata*. *Argentina tricrenulata* was widespread in the Northern Hemisphere, known from Denmark, England, West Greenland and Ellesmere Island and it resembles *A. antarctica* closest of all fossil argentinid otoliths. *Argentina antarctica* differs from *A. tricrenulata* in being more elongate (OL:OH = 1.6 vs. 1.4–1.5 in specimens of comparable size), showing a more rounded postdorsal region (vs. sharp postdorsal angle) and intense crenulation of the rims.

*Argentina longirostris* Schwarzhans, 2003 is clearly more elongate than *A. antarctica* (OL:OH = 1.75–1.95 in specimens larger than 2 mm in length, vs. 1.6) and shows a caudal tip nearly touching the posterior rim of the otolith and connected to it via a postcaudal depression.

*Protargentinolithus extenuatus* (Stinton, 1966). The holotype refigured by Nolf (2013) shows that *Protargentinolithus procerus* Schwarzhans, 2003 is a junior synonym of *Argentina extenuata*. *Protargentinolithus* otoliths grow to very large sizes for an argentinid, up to 8 mm in length, and are characterized by a short, robust rostrum and a short ostium.

*Protargentinolithus erraticus* (Rödel, 1930). Following the selection of a well-preserved lectotype by Nolf (2013), this name replaces *P. balticus* (Rödel, 1930), which is based on a rather poorly preserved specimen. In addition to the short, massive rostrum, *P. erraticus* is further characterized by a low, regularly rounded postdorsal rim.

Nowadays, argentinid species are primarily Northern Hemisphere fishes from temperate to tropical realms, but there are also a few southern temperate species found around Chile, Australia and New Zealand. When Schwarzhans (1980) described the first fossil argentinid record from the early Miocene of New Zealand (*Argentina subfrigida* Schwarzhans, 1980) he assumed that this species would represent an invader species from northern temperate seas. The record of *A. antarctica*, however, shows that the presence of argentinid fishes in the southern temperate seas dates back much earlier into Eocene times, indicating that argentinids may have had a bipolar temperate distribution pattern during the Palaeogene. They are lacking from the warmer shallow water deposits of the European Palaeogene.

Order **Aulopiformes** Rosen, 1973

Family **Paraulopidae** Sato & Nakabo, 2002

Genus ***Paraulopus*** Sato & Nakabo, 2002

***Paraulopus* sp.**

(Fig. 3J, K)

#### Material

One large, rather strongly eroded specimen NRM-PZ P.15965.

#### Occurrence

Site IAA 2/95, La Meseta Formation, Seymour Island, Antarctica.

#### Description

The single otolith of 6.6 mm in length represents a large and diagnostically mature specimen, but unfortunately is strongly leached and eroded on the surface prohibiting a specific identification. It is elongate (OL:OH = 1.95), thin (OH:OT = 3.2) and with a convex inner and a flat outer face. The ventral rim is regularly curved, the dorsal rim undulating, with a somewhat pronounced postdorsal portion. The narrow sulcus is located almost diagonally on the inner face with a long, narrow cauda about 1.5 times the length of the slightly widened ostium, which opens to the anterodorsal margin.

#### Remarks

The appearance is typical for *Paraulopus* otoliths (and Chlorophthalmidae, where these fishes have been placed prior to their rearrangement) and similar otoliths have been reported as widespread in Late Cretaceous and Palaeogene sediments on a worldwide scale. *Paraulopus postangulatus* (Nolf & Dockery, 1993) and *P. novellus* Schwarzhans, 2012 were recorded from the Paleocene of the northern Atlantic basins, the latter resembling the Antarctic specimen quite closely except for the downturned caudal tip. *Paraulopus integer* (Schwarzhans, 1980) from the Eocene of New Zealand and South Australia resembles in the very narrow cauda, but is more compressed.

Order **Myctophiformes** Regan, 1911

Family **Myctophidae** Gill, 1893

Genus ***Diaphus*** Eigenmann & Eigenmann, 1890

***Diaphus*? *marambionis*** sp. nov.

(Figs 3D–I, 6B, C)

#### Material

Holotype: NRM-PZ P.15966 (Figs 3D–F, 6B, C). Paratypes: two specimens NRM-PZ P.15967 (Fig. 3G–I). Other material: two fragmentary specimens NRM-PZ P.15968.

#### Occurrence

Telm 5 unit; ‘*Natica* horizon’, *Cucullea* I member, La Meseta Formation, late Ypresian, early Eocene. Site IAA 2/95, Seymour Island, Antarctica.

#### Etymology

Named after Marambio, the Argentinian research station on Seymour Island.

#### Diagnosis

OL:OH about 1.3. Ventral rim shallow. Dorsal rim regularly rounded, slightly depressed postdorsally. Rostrum about 10% of OL. OCL:CCL = 1.5. Inner face convex. Dorsal margin of ostium curved. Caudal pseudocolliculum well developed.

#### Description

One well-preserved and four incompletely preserved or partly encrusted otoliths; moderately thick and up to nearly 6 mm in length (holotype 5.2 mm long). OH:OT = 3.5–4.0. Dorsal rim rather regularly curved, slightly undulating, somewhat depressed postdorsally and regularly inclined predorsally, sometimes with slight postdorsal angle. Ventral rim shallow, slightly undulating, with (eroded) denticles or protuberances indicated by up to eight grooves seen in ventral view (Fig. 3E, G). Rostrum slightly projecting, blunt, about 10% of OL. Posterior rim rounded, with its tip above caudal tip.

Inner face markedly convex, with moderately wide, median, shallow sulcus. Ostium about 1.5 times the length of cauda and equally wide, its dorsal margin slightly curved; cauda slightly bent upwards, terminating at moderate distance from posterior rim of otolith, its colliculum narrower than ostial colliculum; caudal pseudocolliculum well developed. Dorsal depression wide; ventral field with distinct ventral furrow moderately close to ventral rim of otolith and few faint radial furrows underpinning the marginal crenulation or inter-denticle incisions. Outer face flat to slightly concave, rather smooth, with faint postcentral umbo.

#### Remarks

The degree of variability appears to be relatively low in this species and confined to minor variations of the expression of the postdorsal depression and the thickness of the otoliths.

A number of myctophid otoliths have been described from the Eocene strata of south-west France (Nolf 1988) and Australia (Schwarzhans 1985) as well as the early Oligocene of Italy (Nolf & Steurbaut 1988) and have mostly been placed in the genus *Diaphus*. Most of the Eocene species are characterized by compressed roundish otoliths without denticles at the ventral rim or some degree of crenulation and we consider the majority of those to represent the fossil skeleton-based genus *Eomyctophum* (otoliths *in situ* figured by Schwarzhans 1985), while those of the early Oligocene and a few from the late Eocene exhibit all the characters considered to be diagnostic for modern *Diaphus* otoliths (Schwarzhans 2013). These characters include the shallow ventral rim with denticles, the shape of the dorsal rim with the depressed postdorsal region, the dorsally shifted tip of the posterior rim and the proportions of the sulcus.

The otoliths of *D.*? *marambionis* sp. nov. share those diagnostic features with otoliths of extant species of the genus *Diaphus* and its close relative *Lobianchia*, except for the indistinctly preserved denticles at the ventral rim, and thus represent the earliest record of the *Diaphus*/*Lobianchia* lineage known to date. The two genera are difficult to distinguish by means of otoliths (Schwarzhans 2013) and it is quite possible that *D.*? *marambionis* could represent a taxon predating the dichotomy of the two extant genera. We therefore consider the placement of *D.*? *marambionis* as preliminary until a more detailed review of Palaeogene myctophid otoliths has been performed or more otoliths *in situ* have been retrieved. *Diaphus*? *marambionis* is also remarkable for achieving a considerable size, though not quite reaching the size of the largest early Oligocene species of *Diaphus*.

Order **Gadiformes** Goodrich, 1909

Family **Moridae** Berg, 1940

Genus ***Tripterophycis*** Boulenger, 1902

***Tripterophycis immutatus*** Schwarzhans, 1980

(Fig. 4A, B)

1980 *Tripterophycis immutatus* Schwarzhans: fig. 214.

1985 *Tripterophycis immutatus* Schwarzhans 1980; Schwarzhans: 22, figs 33–35.

#### Material

One large, posteriorly eroded specimen, NRM-PZ P.15969, Site IAA 2/95, La Meseta Formation, Seymour Island, Antarctica.

#### Description

A single, rather large otolith of about 6.7 mm in length. The specimen displays features characteristic for morid otoliths such as the thick appearance where otolith height and thickness is very similar, the flat inner face with the very peculiar sulcus with its flat, oval ostial colliculum and the ridge-like, sharp caudal colliculum sitting in a very depressed, deep cauda. The rear part of the thin, ridge-like caudal colliculum and the posterior tip of the otolith have been broken off in this particular specimen, as is often the case with morid otoliths.

#### Remarks

The single otolith is about twice the size of the otoliths hitherto recorded from South Australia and New Zealand and differs somewhat in being less elongate (OL:OH = 2.7 vs. 3.1–3.3), although this may be exaggerated by the lack of the rear tip of the otolith. We consider this difference as well as few minor variations in the thickness of the dorsal and ventral rims as an expression of ontogenetic changes.

The genus *Tripterophycis* now lives on the continental slope, like most morids, of the Southern Ocean. Its otoliths resemble the much more species-rich tropical to temperate genus *Physiculus* distributed through all oceans, differing primarily by the lack of a predorsal lobe and a bulge of the posterodorsal rim situated well behind the posterior tip of the crista superior. The Eocene *T. immutatus* likewise appears to have been a species with a circum-Southern Ocean distribution. A second, more elongate species is known from the Eocene of South Australia – *T. elongatissimus* Schwarzhans, 1985.

Family **Merlucciidae** Rafinesque, 1815

Subfamily **Macruroninae** Regan, 1903

Genus ***Macruronus*** Günther, 1873

***Macruronus eastmani*** sp. nov.

(Figs 4C–F, 6D–F)

1985 *Macruronus* sp. Schwarzhans: 25, figs 40–42.

#### Material

Holotype: NRM-PZ P.15970 (Figs 4C–E, 6D–F). Paratypes: two specimens, NRM-PZ P.15971 (Fig. 4F). Other material: six fragmentary specimens, NRM-PZ P.15972.

#### Occurrence

Telm 5 unit; ‘*Natica* horizon’, *Cucullea* I member, La Meseta Formation, late Ypresian, early Eocene. Site IAA 1/90, Seymour Island, Antarctica.

#### Etymology

Named in honour of Joseph T. Eastman (Athens, Ohio, USA) in recognition of his contribution to the knowledge of fossil Antarctic fishes. Together with Lance Grande, he was also the first to recognize the presence of fossil gadiforms in the Eocene of the La Meseta Formation.

#### Diagnosis

OL:OH = 1.85–1.95. Ventral rim regularly and moderately deeply curved. All rims and outer face intensely crenulated. CCL:OCL = 1.3. Collum rather wide, with distinct pseudocolliculum.

#### Description

Moderately large and thin otoliths up to at least 8 mm in length (holotype 6 mm long) and rather compressed for a species of the genus *Macruronus*. OH:OT = 3.7–4.0. Dorsal rim divided into three equally long stretches with broad, obtuse predorsal and rounded postdorsal angles at joints, pre- and postdorsal rims regularly inclined, mediodorsal rim flat to slightly concave, slightly backward inclined; predorsal angle consequently highest point on dorsal rim. Ventral rim regularly curved and moderately deep, deepest just anterior of collum. Anterior tip rounded, at level of ostium; posterior tip slightly projecting, rounded. All rims intensely crenulated, medioventral rim the least.

Inner face convex, bent along horizontal axis, with slightly supramedian positioned, moderately wide and shallow sulcus terminating close to anterior and posterior tips of otolith and almost joining them. Sulcus curved, deepest at collum, with ostium only slightly shorter than cauda. Ostial and caudal colliculi well marked, shallow; ostial colliculum anteriorly not reduced; caudal colliculum with slightly bent dorsal margin. Collum moderately wide, with distinct pseudocolliculum. Dorsal field with many long radial furrows, partly reaching into sulcus, and small dorsal depression; ventral field with distinct, thin ventral furrow moderately close to ventral rim of otolith, several faint radial furrows, mostly not extending across ventral furrow. Outer face concave with many long radial furrows coalescing in a shallow central ridge.

#### Remarks

The first otoliths of this species were recovered from the late Eocene of South Australia (Schwarzhans 1985), but they were too poorly preserved to allow a specific identification at the time. The specimens from Seymour Island clearly represent the same species characterized by the relatively compressed shape when compared to other species of the genus, which always have an OL:OH well above 2.0, the deeper ventral rim, the rather long ostium (CCL:OCL = 1.3) and the presence of a pseudocolliculum in the collum. *Macruronus eastmani* is the earliest known species of the genus, and the diagnostic characters are all considered plesiomorphic, except possibly for the pseudocolliculum.

Now, *Macruronus* is a typical endemic gadiform of the temperate Southern Ocean living above the lower shelf and the upper slope. It is identified as an old merlucciid lineage and it appears to have always been geographically bound to the same general area through its evolution during the Palaeogene and Neogene (Schwarzhans 1980, 1985). In New Zealand, *Macruronus* is first recorded in the late Oligocene, after the establishment of the circum-Antarctic deep water current (Schwarzhans 1980). During the Eocene, the Merlucciidae was represented by the extinct otolith-based genus *Macrurulus* Schwarzhans, 1980 in New Zealand.

Family **Gadidae** Rafinesque, 1810

Genus ***Palimphemus*** Kner, 1862

#### Remarks

The fossil otolith-based genus *Colliolus* Gaemers & Schwarzhans, 1973, commonly recorded from the Oligocene and Miocene of the North Sea Basin, has recently been synonymized with the skeleton-based *Palimphemus* after otoliths *in situ* were found in a specimen from Poland (Schwarzhans 2014).

***Palimphemus seymourensis*** sp. nov.

(Figs 4G–M, 6G–I)

#### Material

Holotype: NRM-PZ P.15973 (Figs 4G–I, 6G–I). Paratypes: six specimens, NRM-PZ P.15974–15975 (Fig. 4J–M).

#### Occurrence

Telm 5 unit; ‘*Natica* horizon’, *Cucullea* I member, La Meseta Formation, late Ypresian, early Eocene. Site IAA 2/95, Seymour Island, Antarctica.

#### Etymology

Named after the type locality Seymour Island in Antarctica.

#### Diagnosis

Outline droplet-shaped with broadly rounded anterior rim and slightly elevated predorsal region. OL:OH = 1.8–1.9. Inner face moderately convex. Sulcus narrow, with moderately widened collum and distinct, but short pseudocolliculum. CaL:OsL (measured from centre of collum) = 1.2–1.35. Colliculi often reduced towards outer margin, particularly ostial colliculum. Ventral furrow well developed, close to ventral rim of otolith.

#### Description

Moderately elongate and moderately thick droplet-shaped otoliths of up to 4.5 mm in length (holotype 4.5 mm long). OH:OT = 2.1–2.5. Dorsal rim highest anteriorly above ostial collum in a broadly rounded predorsal angle, somewhat undulating, posteriorly regularly declining without prominent angle. Ventral rim smooth, regularly curved, deepest below rear part of ostium. Anterior rim broadly rounded, with its tip mostly below ostium; posterior rim tapering with rounded tip at level of cauda.

Inner face moderately convex with slightly supramedian, narrow and rather shallow sulcus. Ostium slightly shorter than cauda and slightly narrower. Ostial and caudal colliculi well marked, with relatively wide collum in between and often reduced towards anterior and posterior tips of otolith. Collum somewhat narrowed from ventral with short but distinct pseudocolliculum. Dorsal depression narrow, with indistinct dorsal margin; ventral furrow well developed and close to ventral rim of otolith. Outer face slightly convex to nearly flat, with some radial furrows coalescing in a shallow umbo opposing the collum of the inner face.

#### Remarks

Otoliths of *P. seymourensis* vary slightly in the expression of the ornamentation of the dorsal rim and the outer face, irrespective of the size of the otoliths. Small specimens often show somewhat reduced colliculi terminating more distantly from the anterior and posterior otolith rims (Fig. 4K, M) than is the case in some larger ones (Fig. 4J).

*Palimphemus seymourensis* represents the first record of a gadid from Antarctica and the Southern Hemisphere, except for the occurrence of *Gadiculus antipodus* Schwarzhans, 1980 in the early Miocene of New Zealand and the Recent *Micromesistius australis* off the southern tip of South America and off New Zealand. In the Northern Hemisphere, *Palimphemus* is well recorded since the early Oligocene. From these species, such as *P. brevicollum* (Gaemers, 1994) (in Schwarzhans 1994), the new species differs in the relatively thin appearance, the wider collum with a longer pseudocolliculum, and the low index CaL:OsL (1.2–1.35 vs. 1.7–2.0). Early species of the related genus *Trisopterus* from the Oligocene of Europe show a narrow collum and no pseudocolliculum (Schwarzhans 1994). The Paleocene and early Eocene of Denmark, England and Greenland have yielded species of the related fossil otolith-based genus *Protocolliolus* Gaemers, 1976, which differ from *Palimphemus* otoliths in the slightly narrower collum without a pseudocolliculum, while the index CaL:OsL is similarly low as in *P. seymourensis*.

The discovery of *P. seymourensis* in the early Eocene of Antarctica now represents the earliest record of the genus. It further documents that the Gadidae have had a bipolar temperate distribution in the Palaeogene as opposed to the clearly temperate Northern Hemisphere dominance of today.

Family **Macrouridae** Bonaparte, 1832

Subfamily **Macrourinae** Bonaparte, 1832

Genus ***Coelorinchus*** Giorna, 1809

***Coelorinchus balushkini*** sp. nov.

(Figs 4S–U, 6M, N)

#### Material

Holotype: NRM-PZ P.15976 (Figs 4S–U, 6M, N). Paratypes: two specimens, NRM-PZ P.15977.

#### Occurrence

Telm 5 unit; ‘*Natica* horizon’, *Cucullea* I member, La Meseta Formation, late Ypresian, early Eocene. Site IAA 1/90, Seymour Island, Antarctica.

#### Etymology

Named in honour of A. V. Balushkin (St Petersburg, Russia) in recognition of his contribution to the knowledge of Antarctic fishes, fossil and Recent.

#### Diagnosis

OL:OH = 1.7. Dorsal rim with marked predorsal angle. Ventral rim moderately deeply curved, deepest anteriorly, flattened at deepest point. CCL:OCL = 1.6; CaL:OsL = 1.45. Collum moderately wide, with faint pseudocolliculum.

#### Description

Moderately compressed and moderately thin otoliths of up to at least 6 mm in length (holotype 6.0 mm long). OH:OT = 2.7. Dorsal rim highest anteriorly above ostial collum with rather sharp predorsal angle, rim somewhat undulating, posteriorly regularly declining without prominent angle. Ventral rim deep, deepest below ostium and deepest area flattened. Anterior rim blunt, with obtuse angular tip at level of ostium; posterior rim tapering, rounded, with tip at level of cauda.

Inner face moderately convex with distinctly supramedian, moderately narrow and shallow sulcus. Ostium distinctly shorter than cauda and narrower. Ostial and caudal colliculi well marked, with relatively wide collum in between; ostial colliculum usually reduced towards anterior tip of otolith. Collum narrowed from ventral with short, indistinct pseudocolliculum. Dorsal depression narrow, indistinct; no ventral furrow; dorsal and ventral fields with some radial furrows from the marginal crenulation. Outer face almost flat, with many radial furrows coalescing in a shallow umbo opposing the collum of the inner face.

#### Remarks

A typical species of the genus *Coelorinchus* characterized by its rather compressed outline, the high dorsal rim and the presence of a small and short pseudocolliculum. It resembles *C. buonaiutoi* Schwarzhans, 1985 from the late Eocene of South Australia, but differs in the well-developed and sharp predorsal angle (vs. depressed) and the absence of a ventral furrow on the ventral field (vs. distinctly present).

***Coelorinchus nordenskjoeldi*** sp. nov.

(Figs 4N–R, 6J–L)

#### Material

Holotype: NRM-PZ P.15978 (Figs 4N–P, 6J–L). Paratypes: eight specimens, NRM-PZ P.15979–15980 (Fig. 4Q, R). Other material: 11 eroded or fragmentary specimens, NRM-PZ P.15981–15982.

#### Occurrence

Telm 5 unit; ‘*Natica* horizon’, *Cucullea* I member, La Meseta Formation, late Ypresian, early Eocene. Site IAA 1/90, Seymour Island, Antarctica.

#### Etymology

Named in honour of Dr Otto Nordenskjöld, leader of the Swedish South Polar Expedition 1901–1903.

#### Diagnosis

OL:OH = 1.9–2.0. Dorsal rim with broad predorsal angle. Ventral rim rather shallow, deepest anteriorly, sometimes flattened at deepest point. CCL:OCL = 1.3; CaL:OsL = 1.3–1.45. Collum narrow, ventrally indented, without pseudocolliculum.

#### Description

Moderately elongate and moderately thin otoliths of up to at least 6 mm in length (holotype 5.7 mm long). OH:OT = 2.6–3.0. Dorsal rim highest anteriorly above ostial collum with broad predorsal angle, rim somewhat undulating, posteriorly regularly declining with very broad, indistinct postdorsal angle near end of cauda. Ventral rim moderately shallow, deepest below ostium and deepest area flattened in large specimens. Anterior rim with obtuse angular tip at level of ostium; posterior rim tapering, somewhat projecting, rounded, with tip at level of cauda.

Inner face slightly convex with distinctly supramedian, moderately narrow and shallow sulcus. Ostium distinctly shorter than cauda. Ostial and caudal colliculi well marked, with narrow, ventrally indented collum; no pseudocolliculum. Dorsal depression narrow, indistinct; ventral furrow feeble, close to ventral rim of otolith; few radial furrows along rims, diminishing in large specimens. Outer face almost flat, with many radial furrows coalescing at centre of outer face without umbo.

#### Remarks

*Coelorinchus nordenskjoeldi* was the most common species at the collection site. Yet it shows little variability, being mainly restricted to the expression of the ornamentation of the otolith rims and the outer face.

*Coelorinchus nordenskjoeldi* is readily distinguished from the co-occurring *C. balushkini* through its more elongate shape (OL:OH = 1.9–2.0 vs. 1.7) and narrow, indented collum without pseudocolliculum (vs. moderately wide collum and with pseudocolliculum). There are no comparable species from other Eocene locations of the Southern Ocean.

***Coelorinchus* sp.**

(Fig. 4V, W)

#### Material

Three large, strongly eroded and incomplete specimens, NRM-PZ P.15911, P.15983.

#### Occurrence

Site IAA 1/90, La Meseta Formation, Seymour Island, Antarctica.

#### Description

These large otoliths up to at least 9 mm in length are readily recognized by their strongly expanded predorsal lobe and the strongly convex inner face and strongly concave outer face.

#### Remarks

They represent no doubt a third, yet undescribed species, but none of the specimens are well-enough preserved to serve as the type specimen.

Order **Ophidiiformes** Berg, 1937

Suborder **Ophidioidei** Berg, 1937

Family **Ophidiidae** Rafinesque, 1810

Subfamily **Neobythitinae** Radcliffe, 1913

Genus ***Hoplobrotula*** Gill, 1863

***Hoplobrotula*? *antipoda*** sp. nov.

(Figs 5A–C, 6O, P)

#### Material

Holotype: NRM-PZ P.15984 (Figs 5A, B, 6O, P). Paratype: NRM-PZ P.15985 (Fig. 5C).

#### Occurrence

Telm 5 unit; ‘*Natica* horizon’, *Cucullea* I member, La Meseta Formation, late Ypresian, early Eocene. Site IAA 1/90, Seymour Island, Antarctica.

#### Etymology

Named for its occurrence on the opposite side of the globe when compared to other Eocene species of the genus, known from Europe.

#### Diagnosis

OL:OH = 1.75–1.95. Dorsal rim with low, broad predorsal lobe. OCL:CCL = 2.5–2.75. Sulcus and particularly ostium very narrow.

#### Description

Relatively small, elongate and thin otoliths up to 4 mm in length (holotype 3.9 mm long). OH:OT = 2.6. Dorsal rim rather regularly and gently curved with broad, not much expanded predorsal lobe. Ventral rim regularly curved, deepest slightly in front of its middle. Anterior tip tapering, inferior, below level of ostium; posterior tip narrow, tapering, at level of cauda. All rims smooth.

Inner face moderately convex, smooth, with very narrow, shallow, slightly supramedian sulcus. Ostium very narrow, about 2.5 to 3 times the length of cauda, reaching close to anterior rim of otolith; cauda short, slightly deepened, with rounded tip, terminating at some distance from posterior rim of otolith. Ventral margin of sulcus distinctly indented at ostial–caudal joint. Dorsal depression indistinct, small; ventral furrow faint, fading towards posterior, close to ventral rim of otolith. Outer face slightly convex, less than inner face, smooth.

#### Remarks

*Hoplobrotula*? *antipoda* is a typical representative of the many small ophidiid otoliths, which are so common in the Palaeogene. It clearly differs from the species described from the Eocene of Europe such as *Hoplobrotula biscaica* (Sulc, 1932), *H. greenwoodi* Nolf, 1980, *H. robusta* Nolf, 1980, *H. melrosensis* (Dante & Frizzell, 1965), *H. waltoni* (Schubert, 1916), as well as *Ampheristus toliapicus* König, 1825 and *A. lerichei* (Stinton & Nolf, 1970) (see Nolf 1980, 2013 for figures) by the very narrow and long ostium and the low predorsal lobe. Otoliths of *Hoplobrotuloides bartonensis* (Schubert, 1916) are similarly elongate, but differ in the absence of a predorsal lobe, the flat ventral rim and the broadly expanded posterior tip (see Nolf 1980; Schwarzhans 1981). It is possible that *H.*? *antipoda* represents yet another extinct genus related to the extant genus *Hoplobrotula* and the fossil *Ampheristus*, but a thorough review of the group would be required first. The only other species from the Eocene of the Southern Hemisphere is *Ampheristus sinuocaudatus* Schwarzhans, 1980 from New Zealand, which, however, shows a cauda nearly of the length of the ostium and distinctly deepened.

Otolith data suggest that ophidiiforms were one of the dominant teleost groups in the warm shallow seas, for instance in Europe during the Palaeogene. Temperate seas, such as probably existed in the middle Paleocene of Denmark or the Eocene of New Zealand and South Australia, were comparatively sparse in ophidiiform otoliths and have also yielded far fewer species. This observation is again corroborated by the finds in the Eocene of Antarctica.

Order **Beryciformes** Regan, 1909

Suborder **Berycoidei** Regan, 1909

Family **Berycidae** Lowe, 1839

Genus ***Centroberyx*** Gill, 1862

***Centroberyx* sp.**

(Fig. 5H)

#### Material

A single, strongly eroded specimen, NRM-PZ P.15986.

#### Occurrence

Site IAA 1/90, La Meseta Formation, Seymour Island, Antarctica.

#### Remarks

Despite its very poor preservation expressed in relief reversal of the otolith rims, this otolith is readily recognizable as a representative of the genus *Centroberyx* by its outline in combination with the very large and wide rostrum and the short, somewhat upward trending cauda. *Centroberyx* otoliths are common in the Late Cretaceous and Palaeogene of many locations (Nolf 2013).

Family **Trachichthyidae** Bleeker, 1856

Genus ***Notoberyx*** gen. nov.

#### Type species

*Notoberyx cionei* sp. nov.

#### Etymology

From *notos* (Greek) = southern winds, and the genus name *Beryx*, referring to the southern occurrence of the type species.

#### Diagnosis

Very high-bodied otoliths with a ratio OL:OH between 0.7 and 0.85. Dorsal rim expanded across entire length. Ventral rim deep. Rostrum short, blunt, not much extending beyond level of antirostrum. Ostium short, narrow, only slightly widened ventrally. Cauda long, distinctly turned upwards towards its tip. CaL:OsL = 1.3–1.7.

#### Remarks

The general appearance of *Notoberyx* is typical for trachichthyid otoliths with the deep ventral rim and the upward bent cauda. They are, however, readily distinguished from the many other otolith-based genera of the family known from the Late Cretaceous and Palaeogene by the high body and the short and narrow ostium combined with a short rostrum.

#### Included species

The type species *N. cionei* sp. nov. described below from the Eocene of Antarctica, and *N. madseni* (Schwarzhans, 2007), originally described as ‘genus *Caproidarum*’ *madseni* from the early Eocene of Denmark.

***Notoberyx cionei*** sp. nov.

(Figs 5D–G, 6O)

#### Material

Holotype: NRM-PZ P.15987 (Figs 5D–F, 6O). Paratypes: two specimens, NRM-PZ P.15988–15989 (Fig. 5G).

#### Occurrence

Telm 5 unit; ‘*Natica* horizon’, *Cucullea* I member, La Meseta Formation, late Ypresian, early Eocene. Site IAA 2/95, Seymour Island, Antarctica.

#### Etymology

Named in honour of Alberto Cione (La Plata, Argentina) for his many contributions to the knowledge of fossil fishes from South America and Antarctica.

#### Diagnosis

OL:OH = 0.7–0.75. Dorsal rim high, broad; ventral rim deep, broad. Rostrum short, blunt, about equal length of antirostrum. CaL:OsL = 1.4–1.5. Ostium narrow, only slightly wider than cauda; cauda distinctly turned upwards.

#### Description

Large, robust, high-bodied, oval otoliths up to about 6 mm in length (holotype 5.8 mm long). OL:OT = 3.0. Dorsal rim high, much expanded and broad, undulating; rounded pre- and postdorsal angles close to anterior and posterior limits of dorsal rim, postdorsal angle usually somewhat pronounced. Ventral rim deep, nearly as broad and expanded as dorsal rim, but more gently curved and smooth. Anterior rim blunt, nearly vertically cut, with very short and massive rostrum not reaching beyond length of antirostrum; with broad, shallow excisura in between. Posterior rim likewise blunt and nearly vertically cut, its tip shifted dorsally above level of caudal tip.

Inner face bent along the horizontal axis, nearly straight in vertical direction. Sulcus slightly supramedian, wide, moderately deep. Ostium anteriorly opened, slightly deepened, distinctly shorter than cauda and only slightly wider. OsH:CaH = 1.15–1.3. Cauda long, bent upwards, terminating close to posterior rim of otolith, its colliculum with well-marked ventral rim. Dorsal depression wide, large, well defined towards sulcus; dorsal field with some short radial furrows near the dorsal margin. Ventral field smooth, with distinct ventral furrow very close to ventral rim of otolith. Outer face with broad central umbo.

#### Remarks

These highly diagnostic otoliths differs from the only congener, *N. madseni* from the Northern Hemisphere, in being even more compressed (OL:OH = 0.7–0.75 vs. 0.8–0.85), the ostium being slightly wider than the cauda (vs. not being wider at all) and the absence of the peculiar deepening of the rear portion of the cauda as observed in *N. madseni*. The latter character could possibly support placing *N. madseni* in a genus of its own, once more species with this morphological pattern have become known to support a further taxonomic division.

The two species here attributed to *Notoberyx* show a bipolar temperate distribution with one species each in the early Eocene of Antarctica and Denmark, subject, however, to further finds of these highly diagnostic otoliths. The Danish Eocene otoliths of *N. madseni* were exclusively found in burrow concretions of stomatopods, apparently indicating that these fishes represented their preferred prey.

Order **Perciformes** Bleeker, 1859

Suborder **Percoidei** Bleeker, 1859

Family **Cepolidae** Rafinesque, 1810

Genus ***Cepola*** Linnaeus, 1766

***Cepola anderssoni*** sp. nov.

(Figs 5Q, R, 6R)

#### Material

Holotype: NRM-PZ P.15996 (Figs 5Q, R, 6R) (only specimen, broken into two halves during handling).

#### Occurrence

Telm 5 unit; ‘*Natica* horizon’, *Cucullea* I member, La Meseta Formation, late Ypresian, early Eocene. Site IAA 2/95, Seymour Island, Antarctica.

#### Etymology

Named in honour of Johan Gunnar Andersson, a Swedish pioneer of geological research in Antarctica.

#### Diagnosis

OL:OH = 1.85. Dorsal rim shallow, with broadly rounded postdorsal angle. Rostrum sharp; posterior tip less sharp. OsL:CaL = 2.1; OCL:CCL = 2.0. Ostial colliculum reduced anteriorly, terminating at considerable distance from anterior rim of otolith. Outer face flat.

#### Description

Otolith elongate, thin, 4.4 mm long. OH:OT = 3.2. Dorsal rim shallow, gently curving, smooth, with broadly rounded postdorsal angle and highest at postdorsal angle. Ventral shallow, very regularly curving, highest at its middle, smooth. Rostrum long and sharp; dorsal margin of ostial opening regularly ascending without marked excisura or antirostrum. Posterior tip pointed, but considerably less sharply and projecting as rostrum.

Inner face convex with distinctly supramedian positioned narrow sulcus. Sulcus typically S-shaped with cauda being curved upwards from collum prior to terminating with an inferior tip. Ostium twice as long as cauda, slightly wider and narrower. Ostial colliculum not extending to opening of ostium to anterodorsal otolith rim. Collum ascending, wide, with feeble pseudocolliculum. Cauda very short, its small colliculum distinctly deepened. Dorsal depression large, well marked towards sulcus; ventral furrow feeble, running moderately distant from ventral rim of otolith. Outer face flat and smooth.

#### Remarks

Cepolid otoliths are well known from the Eocene of Europe represented by a number of species (see Nolf 2013). Of those, *Cepola excavata* (Stinton, 1978) and *C. bartonensis* Schubert, 1916 resemble the proportions of the otolith (Nolf 2013; Schwarzhans 2007), while other species are more compressed (*C. densa* (Frost, 1934) and *C. robusta* Nolf, 1988). *Cepola anderssoni* differs from all those species by the anteriorly restricted ostial colliculum and the very small cauda. *Cepola yrieuensis* Steurbaut, 1984 from the early Oligocene of south-west France is similar in the short cauda, but differs in the distinct postdorsal angle (vs. rounded postdorsal region) and the ostial colliculum reaching to or close to the anterior rim (vs. anteriorly restricted).

Indeterminate **Acanthopterygii**

#### Remarks

A number of poorly preserved or fragmented otoliths are briefly described below, representing some Acanthopterygian fishes, chiefly of the Percoidei, of which the identification is uncertain and tentative. They are listed for completion of the record, primarily because they are the first of their kind from Antarctica.

**Acanthopterygii indet.**

(Fig. 5I, J)

#### Material

Two specimens: a strongly eroded specimen, NRM-PZ P.15990 (Fig. 5I, J), Site IAA 1/90, La Meseta Formation, Seymour Island, Antarctica; and a broken rear half of an otolith, NRM-PZ P.15991, Site IAA 1/93, Submeseta member (Telm 6), La Meseta Formation, Seymour Island.

#### Remarks

The figured, strongly eroded specimen from Site IAA 1/90 of 3.3 mm in length shows an elongate shape with a massive and long rostrum, a moderately widened ostium and a moderately bent cauda, which is only slightly longer than the ostium. Similar patterns are observed in many extant scorpaenid otoliths, and also many percoids such as serranids (see Nolf 2013). The fragment from the stratigraphically younger IAA 1/93 site stems from a larger specimen and shows a strong, long, inferior spine at the posterior rim, which probably represents a ontogenetic effect.

**Percoidei indet.**

(Fig. 5K, L)

#### Material

A single, broken specimen, NRM-PZ P.15992, Site IAA 1/90, La Meseta Formation, Seymour Island, Antarctica.

#### Remarks

The single, 4 mm long specimen is somewhat eroded and lacks the rostrum, which would have been a diagnostically important character, and resembles serranid otoliths (see Lin & Chang 2012; Nolf 2013; Smale *et al.* 1995).

**Haemulidae? indet.**

(Fig. 5M, N)

#### Material

A single, broken and eroded specimen, NRM-PZ P.15993, Site IAA 1/90, La Meseta Formation, Seymour Island, Antarctica.

#### Remarks

The single, 4.4 mm long specimen is both eroded and damaged at the rostrum and postventral rim. The shallow, spatulate ostium, the steeply bent caudal tip and the strongly convex inner face are all typical for haemulid otoliths. Similar morphologies have been recorded from the European Eocene (Nolf 2013).

**Sparidae? indet.**

(Fig. 5O, P)

#### Material

Three strongly eroded specimens, NRM-PZ P.15994–15995, Sites IAA 1/90 and 2/95, La Meseta Formation, Seymour Island, Antarctica.

#### Remarks

These strongly eroded specimens up to 5 mm in length resemble certain compressed sparid otoliths known from the Palaeogene of Europe (see Schwarzhans 1994; Nolf 2013) and hence are tentatively placed in the Sparidae.

## Discussion

The fossil sites in the La Meseta Formation of Seymour Island, Antarctica, offer a rare insight into Southern Hemisphere warm temperate marine faunas during a time of global climate optimum in the early to middle Eocene. The fish fauna that we describe here by means of otoliths is clearly dominated by the Gadiformes. Two-thirds of all identifiable otoliths represent a total of six different species of gadiforms. Amongst them are groups which today are characteristic of temperate seas, such as the Macruroninae in the Southern Ocean and the Gadidae in the temperate seas of the Northern Hemisphere, but others like the Macrouridae now dominate benthopelagic deep-water fish communities of the world's oceans below 200 m water depth. The occurrence of abundant macrourid otoliths may be unexpected in sediments thought to have been deposited in an estuarine, near-shore environment, but is consistent with the findings in the Paleocene of Denmark (Schwarzhans 2003). Presumably, macrourids also populated shallower seas during the early Palaeogene, or migrated into bathyal environments in the phase of major reorganization of the deep sea during the early Oligocene (Miller 1992; Katz *et al.* 2011). All other groups identified by means of otoliths are relatively rare and contain a variety of taxa most of which again would be expected in sediments of the lower shelf to upper slope. Amongst these are a myctophid (*Diaphus*? *marambionis*), an argentinid, two berycoids, an ophidiid and several, mostly unidentifiable, percoids.

The correspondence of otolith-related data with previously described skeleton-related data from the La Meseta Formation is rather low, except for the Merlucciidae (Eastman & Grande 1989, 1991; Jerzmanska & Swidnicki 1992; Long & Stiwell 2000; Claeson *et al.* 2012), Macrouridae (Kriwet & Hecht 2008) and Berycoidei (Doktor *et al.* 1996) (see Table 1 for comparison of skeleton and otolith-based data). It has often been observed that otolith and skeleton data retrieved from a specific region and geological time interval do not correspond very well in many instances, but rather complement each other (see Nolf 1985, p. 19). The only articulated fish skeletons so far identified represent a clupeid, *Marambionella andreae* Jerzmanska, 1991. Other skeletal teleost remains are represented by mostly sturdy bones or teeth with a high morphological recognition factor of fishes with fragile or small otoliths with a poor fossilization potential or are generally rare in otolith assemblages (Oplegnathidae, Labridae, Trichiuridae), while the obtained otoliths mainly come from fishes with a fragile skeletal composition that easily disintegrate and may be difficult to recognize when found in isolation (Argentinidae, Paraulopidae, Myctophidae and many percoids).

**Table 1.** Comparison of skeletal remains and otoliths obtained from the La Meseta Formation of Seymour Island, Antarctica.

**Skeletal remains**	**Otoliths**
Clupeiformes – Clupeidae	
*Marambionella andreae*	
Argentiniformes – Argentinidae	
	*Argentina antarctica*
Aulopiformes – Paraulopidae	
	*Paraulopus* sp.
Myctophiformes – Myctophidae	
	*Diaphus*? *marambionis*
Gadiformes – Moridae	
	*Tripterophycis immutatus*
Gadiformes – Merlucciidae	
Merlucciidae indet. (skull) *	*Macruronus eastmani*
Merlucciidae indet. (jaw) **	
Gadiformes indet. (scales)	
Gadiformes – Gadidae	
	*Palimphemus seymourensis*
Gadiformes – Macrouridae	
Macrouridae indet. (skull)	*Coelorinchus balushkini*
	*Coelorinchus nordenskjoeldi*
	*Coelorinchus* sp.
Ophidiiformes – Ophidiidae	
	*Hoplobrotula*? *antipoda*
Beryciformes – Berycidae	
	*Centroberyx* sp.
Beryciformes – Trachichthyidae	
Beryciformes indet. (scales)	*Notoberyx cionei*
Acanthopterygii	
	Acanthopterygii indet.
Perciformes –Percoidei	
	Percoidei indet.
Perciformes – Haemulidae	
	Haemulidae? indet.
Perciformes – Sparidae	
	Sparidae? indet.
Perciformes – Oplegnathidae	
Oplegnathidae indet. (beaks)	
Perciformes – Cepolidae	
	*Cepola anderssoni*
Perciformes – Labridae	
Labridae indet. (tooth plate)	
Perciformes – Trichiuridae	
Trichiuridae indet. (teeth)	
Perciformes – Notothenioidei	
Proeleginops grandeastmanorum *	
*Mesetaichthys jerzmanskae* **	

Skeletal data are from Balushkin (1994), Bienkowska-Wasiluk *et al.* (2013), Cione *et al.* (1994), Claeson *et al.* (2012), Doktor *et al.* (1996), Eastman & Grande (1989, 1991), Grande & Eastman (1986), Jerzmanska (1991), Jerzmanska & Swidnicki (1992), Kriwet & Hecht (2008), Long (1991, 1992) and Long & Stiwell (2000); data annotated by asterisks are based on the same specimens: * after Balushkin (1994) and Eastman & Grande (1991); ** after Bienkowska-Wasiluk *et al.* (2013) and Jerzmanska & Swidnicki (1992).

The fish association of the La Meseta Formation, reconstructed from otoliths, reveals three main and possibly unexpected results:

1. None of the Eocene teleost groups have persisted until today in sub-Antarctic waters. Fishes now endemic to the seas surrounding Antarctica are completely missing, notably representatives of the Notothenioidei and the only endemic Antarctic gadiform fish family, Muraenolepididae.
2. There is a high level of shared faunal elements with temperate faunal associations of the Paleocene and Eocene of the Northern Hemisphere, best known from Denmark, England and Greenland. The similarity is not at species level, but at generic and familial levels, for instance the genera *Argentina*, *Palimphemus* (Gadidae) and *Notoberyx* (Trachichthyidae). We interpret this effect as an indication of a former bipolar temperate fish fauna, which has become depleted and/or pushed northwards in the Southern Ocean after effective isolation of the Antarctic Ocean during the late Eocene and the establishment of a boreal climate and freezing of the Antarctic continent (Lear *et al.* 2004; Barrett 2009; Villa *et al.* 2013).

**Figure 7.**
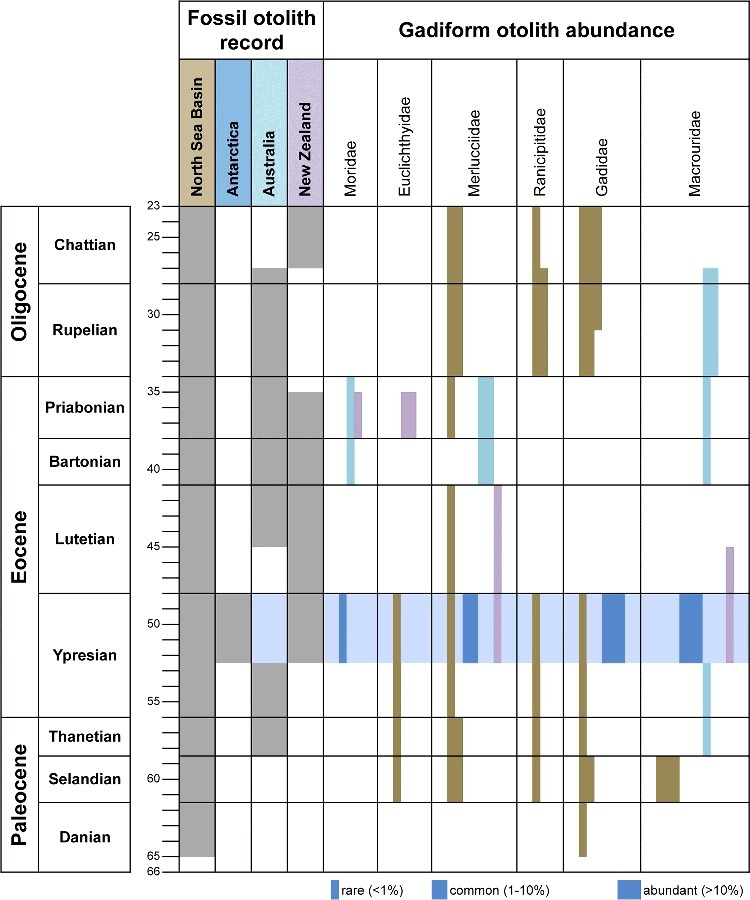
Fossil otolith record in Antarctica, Australia, New Zealand and the North Sea Basin and distribution and estimated abundance of gadiform otoliths (number of species, recognized or inferred, not shown). Ranicipitidae shown in family ranking following Nelson (1994); other families following Nelson (2006). Data compiled and altered from Nolf (2013), Schwarzhans (1980, 1985, 1994, 2003) and Stinton (1965, 1966).

Figure 7 shows the distribution and abundance of gadiform otolith-based taxa estimated for the Palaeogene of Southern Hemisphere locations and the North Sea Basin. The abundance of gadid, macrourid and merlucciid (macruronine) otoliths in the Ypresian of Antarctica contrasts with the comparatively few gadiform records in the Ypresian and Lutetian of Australia, New Zealand and the North Sea Basin. Gadiforms are common in the Paleocene and Oligocene of the North Sea Basin and, to a lesser degree, in the late Eocene of southern Australia. We interpret their abundance in Antarctica during the times of the early Eocene Climate Optimum (EECO) as representing a refugium for temperate fishes. Cooler periods of the Paleocene and particularly from the Oligocene onward show a wider extent and greater abundance of gadiforms in seas of lower latitudes. Certain gadiform otoliths from the Eocene and Paleocene of the North Sea Basin originally described as macrourids by Schwarzhans (2003) have been controversially interpreted as merlucciids by Nolf (2013), without offering an explanation or discussion. We retain the identification of these as macrourids following the reasoning given by Schwarzhans (2003). Another group of problematic otoliths have been described under the fossil otolith-based genera *Gadophycis* Stinton, 1965, *Ensigadus* Gaemers, 1978, or as gadids of an unknown relationship by Nolf (2013). Following the discovery of otoliths *in situ* in the skeleton-based genus *Protobrotula* Daniltchenko, 1960 (Prokofiev 2001; Rozenberg 2003), a genus which has been alternatively interpreted as a gadiform or ophidiiform, we follow the review of Prokofiev (2001) in allocating it with the Ophidiiformes and have therefore excluded all related otolith-based species from our list.

**Figure 8.**
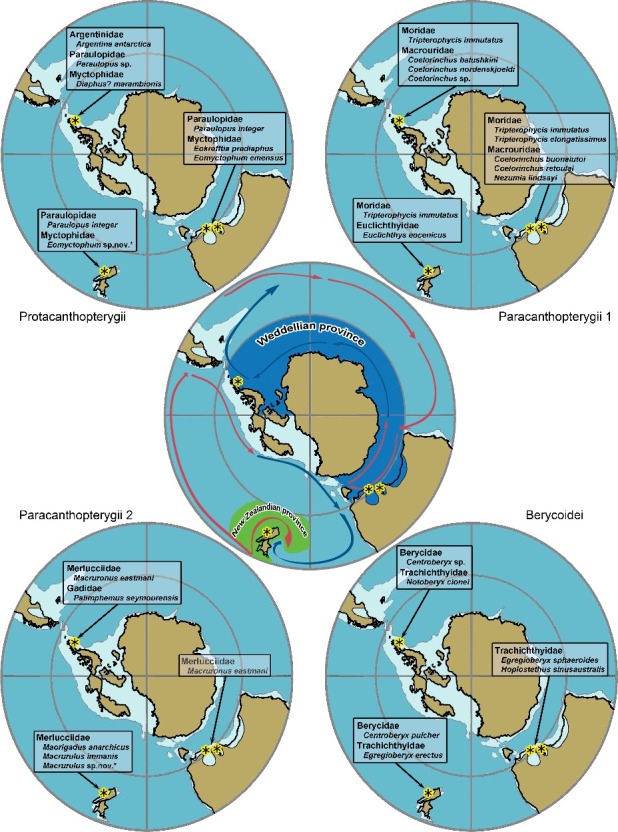
Eocene palaeogeography in south polar projection and the distributions of selected taxa of Protacanthopterygii, Paracanthopterygii and Berycoidei. Regions studied for fossil otoliths are marked by an asterisk (each region may contain multiple locations). Otolith data are compiled from Schwarzhans (1980, 1985); the palaeogeographical reconstruction is based on Reguero *et al.* (2013); the delimitation of the Weddellian bioprovince is based on Zinsmeister (1982); the reconstruction of palaeocurrents is composed from Crame (1999) and Huber *et al.* (2004).

1. The otolith composition shows some similarity with that described from the Eocene of South Australia with at least two shared species (*Macruronus eastmani* and *Tripterophycis immutatus*), and to a lesser degree with that of New Zealand with only one shared species (*Tripterophycis immutatus*). The level of shared higher taxa is otherwise low. We interpret this as an indication of the former southern Atlantic, ‘Weddellian’, bioprovince (Zinsmeister 1982) having been separated from the New Zealandian bioprovince during the Eocene (Fig. 8), and only having become interconnected allowing faunal exchanges after the opening of the strait between Tasmania and Antarctica and the establishment of a circum-Antarctic current (Kennett *et al.* 1972, 1974; Schwarzhans 1980; Barker *et al.* 2007).

Figure 8 summarizes the distribution of certain fish groups in the Southern Ocean during Eocene times based on otoliths. The three areas currently with studied otolith data (Australia, New Zealand and Antarctica) show a relatively similar composition of otolith-based taxa at the genus and familial level, for instance in Paraulopidae, Myctophidae, Beryciformes and, as discussed above, in Gadiformes. Similarities on the genus level are more pronounced between southern Australia and Antarctica than of both to New Zealand (*Paraulopus*, *Tripterophycis*, *Coelorinchus*, *Macruronus*).

The fish fauna as reconstructed from otoliths justifies a more specific comment to the ongoing scientific debate concerning the nature of certain skeletal remains that have been identified as merlucciids by some authors (Eastman & Grande 1989, 1991; Jerzmanska & Swidnicki 1992; Claeson *et al.* 2012) and as notothenioids by others (Balushkin 1994; Bienkowska-Wasiluk *et al.* 2013). The abundance of gadiform otoliths obviously lends support to the merlucciid interpretation. The complete lack of notothenioid otoliths in the La Meseta Formation, however, does not rule out the possibility that certain skeletal remains could represent notothenioids, or that such fishes were present in the sub-Antarctic seas of the time but their otoliths have not yet been discovered, bearing in mind the often complementary nature of otolith and skeleton finds.

The relatively small association of fossil otoliths studied here indicates the potential of additional valuable information that such investigations can deliver. In respect to the endemic Recent marine fish fauna of Antarctica, very little is known about its origin and evolution. The fish fauna from the early Eocene La Meseta Formation obviously predates the onset of this endemic evolution, at least for the most part and certainly as far as the otolith finds are concerned. We believe that more pertinent information for the elucidation of the evolution of the Antarctic fish fauna can be expected from younger Palaeogene and Neogene strata of Antarctica and southernmost South America, which represent periods in the geological history of Antarctica when the climate cooling had progressed further than was the case during the EECO.

## References

[cit0001] Balushkin A. V. (1994). *Proeleginops grandeastmanorum* gen. et sp. nov. (Perciformes, Notothenioidei, Eleginopsidae) from the Late Eocene of Seymour Island (Antarctica) is a fossil notothenioid, not a gadiform. *Journal of Ichthyology*.

[cit0002] Bannikov A. F. (2010). *Fossil vertebrates of Russia and adjacent countries. Fossil Acanthopterygian fishes (Teleostei, Acanthopterygii)*.

[cit0003] Barker P. F., Filippelli G. M., Florindo F., Martin E. E., Scher H. D. (2007). Onset and role of the Antarctic Circumpolar Current. *Deep-Sea Research II*.

[cit0004] Barrett P. (2009). A history of Antarctic Cenozoic glaciation - View from the margin. *Developments in Earth & Environmental Sciences*.

[cit0102] Berg L. S. (1937). A classification of fish-like vertebrates. *Isv. AN SSSR, seri biologika*.

[cit0093] Berg L. S. (1940). Classification of fishes, both recent and fossil. *Trav. Inst. Zool. Acad. Sci. USSR*.

[cit0083] Bertelsen E. (1958). The argentinoid fish *Xenophthalmichthys*
*danae*. *Dana-Report*.

[cit0005] Bienkowska-Wasiluk M., Bonde N., Møller P. R., Gazdzicki A. (2013). Eocene relatives of cod icefishes (Perciformes: Notothenioidei) from Seymour Island, Antarctica. *Geological Quarterly*.

[cit0108] Bleeker P. (1856). Beschrijvingen van nieuwe of weinig bekende vischsoorten van Manado en Makassar, grootendeels verzameld op eene reis naar den Molukschen Archipel in het gevolg van den Gouverneur Generaal Duymaer van Twist. *Acta Societatis Regiae Scientiarum Indo-Nerlandicae*.

[cit0100] Bonaparte C. L. (1832). *Iconografia delle fauna italica per le quattro classi degli animali vertebrati. Tomo III. Pesci*.

[cit0084] Bonaparte C. L. (1846). Catalogo metodico dei pesci europei. *Atti Soc. Ital. Sci. Nat. Milano*.

[cit0094] Boulenger G. A. (1902). Description of a new deep sea gadid fish from South Africa. *Ann. Mag. Nat. Hist.*.

[cit0006] Cione A. L., de las Mercedes Azpelicueta M., Bellwood D. R. (1994). An oplegnathid fish from the Eocene of Antarctica. *Palaeontology*.

[cit0007] Cione A. L., Reguero M. A. (1998). An Eocene basking shark (Lamniformes, Cetorhinidae) from Antarctica. *Antarctic Science*.

[cit0008] Claeson K. M., Eastman J. T., MacPhee R. D. E. (2012). Definitive specimens of Merlucciidae (Gadiformes) from the Eocene James Ross Basin of Isla Marambio (Seymour Island), Antarctica Peninsula. *Antarctic Science*.

[cit0009] Crame J. A. (1999). An evolutionary perspective on marine faunal connections between southernmost South America and Antarctica. *Scientia Marina*.

[cit0010] Del Valle R. A., Elliot D. H., Macdonald D. I. M. (1992). Sedimentary basins on the east flank of the Antarctic Peninsula: proposed nomenclature. *Antarctic Science*.

[cit0011] Dingle R., Lavelle M. (1998). Antarctic Peninsula cryosphere: early Oligocene (c. 30 Ma) initiation and a revised glacial chronology. *Journal of the Geological Society of London*.

[cit0012] Doktor M., Gazdzicki A., Jerzmanska A., Porebski S. J., Zastawniak E. (1996). A plant-and-fish assemblage from the Eocene La Meseta Formation of Seymour Island (Antarctic Peninsula) and its environmental implications. *Palaeontologia Polonica*.

[cit0013] Douglas P. M. J., Affek H. P., Ivany L. C., Houben A. J. P., Sijp W. P., Sluijs A., Schouten S., Pagani M. (2014). Pronounced zonal heterogeneity in Eocene southern high-latitude sea surface temperatures. *Proceedings of the National Academy of Sciences*.

[cit0014] Dutton A. L., Lohmann K., Zinsmeister W. J. (2002). Stable isotope and minor element proxies for Eocene climate of Seymour Island Antarctica. *Paleoceanography*.

[cit0015] Eastman J. T., Grande L. (1989). Evolution of the Antarctic fish fauna with emphasis on the Recent notothenioids. *Geological Society, London, Special Publications*.

[cit0016] Eastman J. T., Grande L. (1991). Late Eocene gadiform (Teleostei) skull from Seymour Island, Antarctic Peninsula. *Antarctic Science*.

[cit0091] Eigenmann C. H., Eigenmann R. S. (1890). Additions to the fauna of San Diego. *Proc. Calif. Acad. Sci.*.

[cit0017] Elliot D. H. (1988). Tectonic setting and evolution of the James Ross Basin, northern Antarctic Peninsula. *Geological Society of America, Memoir*.

[cit0018] Elliot D. H., Trautman T. A., Craddock C. (1982). Lower Tertiary strata on Seymour Island, Antarctic Peninsula. *Antarctic geoscience*.

[cit0019] Gaemers P. A. M. (1976). New gadiform otoliths from the Tertiary of the North Sea Basin and a revision of some fossil and recent species. *Leidse Geologische Mededelingen*.

[cit0020] Gelfo J. N., Reguero M. A., López G. M., Carlini A. A., Ciancio M. R., Chornogubsky L., Bond M., Goin F. J., Tejedor M. F. (2009). Eocene mammals and continental strata from Patagonia and Antarctic Peninsula. *Museum of North Arizona Bulletin*.

[cit0107] Gill T. N. (1862). Remarks on the relations of the genera and other groups of Cuban dishes. *Proc. Acad. Nat. Sci. Philadelphia*.

[cit0104] Gill T. N. (1863). Descriptions of the genera of gadoid and brotulid fishes. *Proc. Acad. Nat. Sci. Philapelphia*.

[cit0090] Gill T. N. (1893). Families and subfamilies of fishes. *Memoirs of the National Academy of Science*.

[cit0101] Giorna M. E. (1809). Mémoire sur les poissons d'espèces nouvelles et de genres nouveaux. Plus: Suite et conclusions du mémoire. *Mem. Acad. Imp. Sci. Lit. eaux-arts*.

[cit0092] Goodrich E. S. (1909). *The Vertebrata Craniata (Cyclostomes and Fishes)*.

[cit0021] Grande L., Eastman J. T. (1986). A review of Antarctic ichthyofaunas in the light of new fossil discoveries. *Palaeontology*.

[cit0097] Günther A. (1873). Pisces. *Zoological Record*.

[cit0022] Hathway B. (2000). Continental rift to back-arc basin: Jurassic–Cretaceous stratigraphical and structural evolution of the Larsen Basin, Antarctic Peninsula. *Journal of the Geological Society of London*.

[cit0023] Huber M., Brinkhuis H., Stickley C. E., Döös K., Sluijs A., Warnaar J., Schellenberg S. A., Williams G. L. (2004). Eocene circulation of the Southern Ocean: Was Antarctica kept warm by subtropical waters. *Paleoceanography*.

[cit0081] Huxley T. H. (1880). On the application of the laws of evolution to the arrangement of the Vertebrata and more particularly of the Mammalia. *Zoological Society of London*.

[cit0024] Ivany L. C., Lohmann K. C., Hasiuk F., Blake D. B., Glass A., Aronson R. B., Moody R. M. (2008). Eocene climate record of a high southern latitude continental shelf: Seymour Island, Antarctica. *Geological Society of American Bulletin*.

[cit0025] Ivany L. C., Van Simaeys S., Domack E. W., Samson S. D. (2006). Evidence for an earliest Oligocene ice sheet on the Antarctic Peninsula. *Geology*.

[cit0026] Janssen A. W. (2012). Validation of holoplanktonic molluscan taxa from the Oligo–Miocene of the Maltese Archipelago, introduced in violation with ICZN regulations. *Cainozoic Research*.

[cit0027] Jerzmanska A. (1991). First articulated teleost fish from the Paleogene of West Antarctica. *Antarctic Science*.

[cit0028] Jerzmanska A., Swidnicki J. (1992). Gadiform remains from the La Meseta Formation (Eocene) of Seymour Island, West Antarctica. *Polish Polar Research*.

[cit0029] Katz M. E., Cramer B. S., Toggweiler J. R., Esmay G., Liu C., Miller K. G., Rosenthal Y., Wade B. S., Wright J. D. (2011). Impact of Antarctic circumpolar current development on late Paleogene ocean structure. *Science*.

[cit0030] Kennett J., Burns R., Andrews J., Churkin M., Davies T., Dumitrica P., Edwards A., Galehouse J., Packham G., van der Lingeren G. (1972). Australian–Antarctic continental drift, paleo-circulation changes and Oligocene deep-sea erosion. *Nature Physical Science*.

[cit0031] Kennett J., Houtz R., Andrews P., Edwards A., Gostin V., Hajos N., Hampton H., Jenkins G., Margolis S., Overshine A., Perch-Nielsen K. (1974). Cenozoic paleo-oceanography in the southwest Pacific Ocean and the development of the circum-Antarctic current. *Initial Reports DSDP*.

[cit0099] Kner R. (1862). Kleinere Beiträge zur Kenntnis der fossilen Fische Österreichs. *Sitzungsberichte der kaiserlichen Akademie der Wissenschaften, Mathematisch-Naturwissenschaftliche Classe, Abt. I*.

[cit0032] Koken E. (1884). Über Fisch-Otolithen, insbesondere über diejenigen der norddeutschen Oligocän-Ablagerungen. *Zeitschrift der deutschen Geologischen Gesellschaft*.

[cit0033] Koken E. (1891). Neue Untersuchungen an Tertiären Fischotolithen II. *Zeitschrift der deutschen Geologischen Gesellschaft*.

[cit0034] Kriwet J., Hecht T. (2008). A review of early gadiform evolution and diversification: first record of a rattail fish skull (Gadiformes, Macrouridae) from the Eocene of Antarctica, with otoliths preserved in situ. *Naturwissenschaften*.

[cit0035] Lear C. H., Rosenthal Y., Coxall H. K., Wilson P. A. (2004). Late Eocene to early Miocene ice sheet dynamics and the global carbon cycle. *Paleoceanography*.

[cit0036] Lin C.-H., Chang C.-W. (2012). Otolith atlas of Taiwan fishes. *NMBA Atlas Series*.

[cit0085] Linnaeus C. (1758). *Systema Naturae, Ed. X. (Systema naturae per regna tria naturae, secundum classes, ordines, genera, species, cum characteribus, differentiis, synonymis, locis. Tomus I. Editio decima, reformata.)*.

[cit0109] Linnaeus C. (1766). *Systema naturae sive regna tria maturae, secundum classes, ordines, genera, species, cum characteribus, differentiis, synonymis, locis. Laurentii Salvii*.

[cit0037] Long D. J. (1991). Fossil cutlassfish (Perciformes: Trichiuridae) teeth from the La Meseta Formation (Eocene) Seymour Island, Antarctic Peninsula. *Paleobios*.

[cit0038] Long D. J. (1992). An Eocene wrasse (Perciformes; Labridae) from Seymour Island. *Antarctic Science*.

[cit0039] Long D. J., Stilwell J. D. (2000). Fish remains from the Eocene of Mount Discovery, East Antarctica. *Antarctic Research Series*.

[cit0106] Lowe R. T. (1839). A supplement to a synopsis of the fishes of Madeira. *Proceedings of the Zoological Society of London*.

[cit0040] Marenssi S. A., Santillana S. N., Rinaldi C. A. (1998). Stratigraphy of the La Meseta Formation (Eocene), Marambio (Seymour) Island, Antarctica. *Asociación Paleontológica Argentina, Publicación Especial*.

[cit0041] Marenssi S. A., Net L. I., Santillana S. N. (2002). Provenance, depositional and palaeogeographic controls on sandstone composition in an incised valley system: the Eocene La Meseta Formation, Seymour Island Antarctica. *Sedimentary Geology*.

[cit0043] Miller K. G., Prothero D. R., Berggren W. A. (1992). Middle Eocene to Oligocene stable isotopes, climate, and deep-water history: the terminal Eocene event?. *Eocene–Oligocene climatic and biotic evolution*.

[cit0044] Montes M., Nozal F., Santillana S., Marenssi S., Olivero E., Serie Cartografica Geocientıfica Antartica (2013). *Mapa Geologico de la isla Marambio (Seymour) Escala 1:20.000 Primera Edicion*.

[cit0082] Müller J. (1846). Über den Bau und die Grenzen der Ganoiden und über das natürliche System der Fische. *Abhandlungen der Königlichen Akademie der Wissenschaften zu Berlin*.

[cit0045] Nelson J. S. (1994). *Fishes of the world*.

[cit0046] Nelson J. S. (2006). *Fishes of the world*.

[cit0047] Nolf D. (1980). Etude monographique des otolithes des Ophidiiformes actuels et révision des espèces fossils (Pisces, Teleostei). *Mededelingen van de Werkgroup voor Tertiaire en Kwartaire Geologie*.

[cit0048] Nolf D. (1985). *Handbook of paleoichthyology, Volume 10, Otolithi piscium*.

[cit0049] Nolf D. (1988). Les otolithes de téléostéens éocènes d'Aquitaine et leur intérêt stratigraphique. *Académie Royale de Belgique, Mémoires de la classe des Sciences*.

[cit0050] Nolf D. (2013). *The diversity of fish otoliths, past and present*.

[cit0051] Nolf D., Dockery D. T. (1993). Fish otoliths from the Matthews Landing Marl Member (Porters Creek Formation), Paleocene of Alabama. *Mississippi Geology*.

[cit0052] Nolf D., Steurbaut E. (1988). Description de la première faune ichthyologique exclusivement bathyale du Tertiare d'Europe: otolithes de l'Oligocène Inférieur du gisement de Pizzocorno, Italie septentrionale. *Bulletin de l'Institute Royal des sciences naturelles de Belgique, Sciences de la terre*.

[cit0053] Prokofiev A. M. (2001). Redescription of *Protobrotula sobijevi* (Daniltshenko, 1953) (Paracanthopterygii, Ophidiiformes) from the Lower Oligocene of the Caucasus. *Journal of Ichthyology*.

[cit0103] Radcliffe L. (1913). Descriptions of seven new genera and thirty-one new species of fishes of the families Brotulidae and Carapidae from the Philippine Islands and the Dutch East Indies. (Scientific results of the Philippine cruise of the Fisheries steamer “Albatross” 1907–1910, No. 24). *Proceedings of the United States National Museum*.

[cit0098] Rafinesque C. S. (1810). *Indice d'ittiologia siciliana; ossia, catalogo metodico dei nomi latini, italiani, e siciliani dei pesci, che si rinvengono in Sicilia disposti secondo un metodo naturale e seguito da un appendice che contiene la descrizione de alcuni nuovi pesci siciliani*.

[cit0095] Rafinesque C. S. (1815). *Analyse de la nature, ou tableau de l'univers et des corps organises*.

[cit0096] Regan C. T. (1903). On the systematic position and classification of the gadoid or anacanthine fishes. *Ann. Mag. Nat. Hist.*.

[cit0105] Regan C. T. (1909). The classification of teleostean fishes. *Ann. Mag. Nat. Hist.*.

[cit0088] Regan C. T. (1911). The anatomy and classification of the teleostean fishes of the order Iniomi. *Ann. Mag. Nat.*.

[cit0054] Reguero M. A., Marenssi S. A., Santillana S. N. (2002). Antarctic Peninsula and Patagonia Paleogene terrestrial environments: biotic and biogeographic relationships. *Palaeogeography, Palaeoclimatology, Palaeoecology*.

[cit0055] Reguero M., Goin F., Acosta Hospitaleche C., Dutra T., Marenssi S. (2013). *Late Cretaceous/Palaeogene west Antarctica terrestrial biota and its intercontinental affinities*.

[cit0056] Rödel H. (1930). Fischotolithen aus Palaeozängeschieben. *Zeitschrift für Geschiebeforschung*.

[cit0086] Rosen D. E., P. H. Greenwood, R. S. Miles, C. Patterson (1973). Interrelationships of higher euteleostean fishes. *Interrelationships of Fishes (Supplement No. 1 to the Zoological Journal of the Linnean Society Vol. 53)*.

[cit0057] Rozenberg A. (2003). *Otolithen mariner Teleosteer aus dem Obereozän/Unteroligozän des Ostparatethys-Nordseebecken-Raumes: Bestandsaufnahme der auf Otolithen basierenden Fischfaunen sowie biostratigraphische und paläobiogeographische Vergleiche und Analyse*.

[cit0058] Sadler P. (1988). Geometry and stratification of uppermost Cretaceous and Palaeogene units of Seymour Island, northern Antarctic Peninsula. *Geological Society of America, Memoir*.

[cit0087] Sato T., Nakabo T. (2002). Paraulopidae and *Paraulopus*, a new family and genus of aulopiform fishes with revised relationships within the order. *Ichthyological Research*.

[cit0059] Schubert R. (1916). Obereozäne Otolithen vom Barton Cliff bei Christchurch (Hampshire). *Jahrbuch der Kaiserlich-Königlichen Geologischen Gesellschaft*.

[cit0060] Schwarzhans W. (1978). Otolith-morphology and its usage for higher systematical units, with special reference to the Myctophiformes s.l. *Mededelingen Werkgroep Tertiaire en Kwartaire Geologie*.

[cit0061] Schwarzhans W. (1980). Die tertiäre Teleosteer-Fauna Neuseelands, rekonstruiert anhand von Otolithen. *Berliner geowissenschaftliche Abhandlungen (A)*.

[cit0062] Schwarzhans W. (1981). Vergleichende morphologische Untersuchungen an rezenten und fossilen Otolithen der Ordnung Ophidiiformes. *Berliner geowissenschaftliche Abhandlungen, (A)*.

[cit0063] Schwarzhans W. (1985). Tertiäre Otolithen aus South Australia und Vict(Australien). *Palaeo Ichthyologica*.

[cit0064] Schwarzhans W. (1986). Fish otoliths from the lower Tertiary of Ellesmere Island. *Canadian Journal of Earth Science*.

[cit0065] Schwarzhans W. (1994). Die Fisch-Otolithen aus dem Oberoligozän der Niederrheinischen Bucht. Systematik, Palökologie, Palaobiogeographie, Biostratigraphie und Otolithen-Zonierung. *Geologisches Jahrbuch, A*.

[cit0066] Schwarzhans W. (2003). Fish otoliths from the Paleocene of Denmark. *Geological Survey of Denmark and Greenland Bulletin*.

[cit0067] Schwarzhans W. (2004). Fish otoliths from the Paleocene (Selandian) of West Greenland. *Meddelser om Grønland*.

[cit0068] Schwarzhans W. (2007). Otoliths from casts from the Eocene Lillebælt Clay Formation of Trelde Næs near Fredericia (Denmark), with remarks on the diet of stomatopods. *Neues Jahrbuch für Geologie und Paläontologie, Abhandlungen*.

[cit0069] Schwarzhans W. (2012). Fish otoliths from the Paleocene of Bavaria (Kressenberg) and Austria (Kroisbach and Oiching-Graben). *Palaeo Ichthyologica*.

[cit0070] Schwarzhans W. (2013). A comparative morphological study of the Recent otoliths of the genera Diaphus, Idiolychnus and Lobianchia (Myctophidae). *Palaeo Ichthyologica*.

[cit0071] Schwarzhans W. (2014). Synonymisation of the skeleton-based Palimphemus anceps Kner, 1862 and the otolith-based Colliolus sculptus (Koken, 1891) (Pisces, Teleostei, Gadidae). *Cainozoic Research*.

[cit0072] Smale M. J., Watson G., Hecht T. (1995). Otolith atlas of southern African marine fishes. *J.L.B. Smith Institute of ichthyology, Ichthyological monographs*.

[cit0073] Stilwell J. D., Zinsmeister W. J. (1992). Molluscan systematics and biostratigraphy: Lower Tertiary La Meseta Formation, Seymour Island, Antarctic Peninsula. *Antarctic Research Series*.

[cit0074] Stinton F. C. (1965). Teleost otoliths from the Lower London Tertiaries. *Senckenbergiana lethaea*.

[cit0075] Stinton F. C., Casier E. (1966). Fish otoliths from the London Clay. *Faune ichthyologique du London Clay*.

[cit0076] Tejedor M. F., Goin F. J., Gelfo J. N., López G. M., Bond M., Carlini A. A., Scillato-Yané G. J., Woodburne M. O., Chornogubsky L., Aragón E., Reguero M. A., Czaplewski N. J., Vincon S., Martin G. M., Ciancio M. R. (2009). New Early Eocene mammalian fauna from western Patagonia, Argentina. *American Museum Novitates*.

[cit0077] Tracey S. (2014). Notes on Nolf's nomenclatural system. Book review – ‘The diversity of fish otoliths, past and present’, by Dirk Nolf. *Cainozoic Research*.

[cit0078] Villa G., Fioroni C., Persico D., Roberts A. P., Florindo F. (2013). Middle Eocene to Late Oligocene Antarctic glaciation/deglaciation and Southern Ocean productivity. *Paleoceanography*.

[cit0079] Weiler W. (1942). Die Otolithen des rheinischen und nordwestdeutschen Tertiärs. *Abhhandlungen des Reichsamt für Bodenforschung*.

[cit0080] Zinsmeister W. J. (1982). Late Cretaceous – Early Tertiary molluscan biogeography of the southern circum-Pacific. *Journal of Paleontology*.

